# Reproductive Biology and Ecology of the Green Mussel *Perna viridis*: A Multidisciplinary Approach

**DOI:** 10.3390/biology8040088

**Published:** 2019-11-15

**Authors:** Md Asaduzzaman, Aysha Rahi Noor, Md Moshiur Rahman, Sumi Akter, Nayeema Ferdausy Hoque, Abrar Shakil, Md Abdul Wahab

**Affiliations:** 1 Department of Marine Bioresource Science, Faculty of Fisheries, Chattogram Veterinary and Animal Sciences University, Khulshi 4225, Chattogram, Bangladesh; shiropa14@gmail.com (A.R.N.); sumicvasu@gmail.com (S.A.); nayeema.ferdausy.hoque@gmail.com (N.F.H.); shakil01852723665@gmail.com (A.S.); 2 Fisheries and Marine Resource Technology Discipline, Khulna University, Khulna-9208, Bangladesh; mrahmankufmrt@gmail.com; 3 WorldFish, Bangladesh and South Asia Office, Banani, Dhaka 1213, Bangladesh; a.wahab@cgiar.org

**Keywords:** green mussel, reproductive biology, gonad development, environmental factors, feeding habits, fatty acid profile, multidisciplinary approach

## Abstract

A multidisciplinary approach was applied to explore deeper knowledge about the reproductive biology and ecology of the green mussel (*Perna viridis*) by interlinking among ecological factors, ingested gut plankton, gonad fatty acid profile, and reproductive traits. Mussels were collected throughout the year from the coastal region of the Bay of Bengal of Bangladesh, and the histological analysis of 242 mussels revealed five stages of gametogenesis with an annual spawning season from January to April. The gonadosomatic index showed a strong correlation with the water quality parameters, ingested gut plankton groups, and gonadal fatty acids, and also displayed prominent effects of seasonality. In all datasets, we performed principal component analysis in which the first two principal components showed that seasonality explained >65% of the variability, and the multivariate spaces of seasonality corresponded to the gonad development. High salinity, nutrients, chlorophyll *a*, and plankton abundance in the water column were positively correlated with gonad development and the spawning of mussels. During the development stage, mussel ingested more plankton, particularly Bacillariophyceae and Pyrrophyceae, which were an important source of increased n-3 polyunsaturated fatty acids content in gonads. Furthermore, gonads accumulated more lipids and saturated fatty acids during the mature and spawning stages, which were probably utilized during embryogenesis and early larval development as energy sources and constituents of the cell membrane. This research provides a model toward a better understanding of reproductive biological traits and how these traits are interrelated with the surrounding environmental variables of the green mussel, which would be helpful in marine aquaculture and the sustainable exploitation of the fishery resources.

## 1. Introduction

Asian green mussel (*Perna viridis*), an estuarine and intertidal bivalve species, is widely distributed in the tropical and subtropical areas of the Indo-Pacific region [1]. It is a large and fast-growing commercially important species, and has demonstrated prodigious culture feasibilities in many countries [2]. The global production of marine mussels (Mytilidae) has been increasing at an average rate of 5% per year since 1990, reaching about 1.1 million tonnes in 2015, of which 85% comes from the Asian countries [3]. Since marine mussels are a sustainable form of protein production, it is expected that the global production of mussels, particularly in Asia, will continue to grow in order to fulfil the protein demand of the growing world population [4]. In Bangladesh, the natural distribution of *P. viridis* is confined to the southeastern coasts, particularly in the Moheshkhali Channel and the Naaf river estuary [5]. Coastal waters are the most productive zones in Bangladesh, and offer an ideal environment for natural growth and recruitment as well as the farming of *P. viridis* [5]. The government of Bangladesh has given high priority for the sustainable management of the wild population and the development of green mussel farming technology to create income opportunities among the impoverished fishers and farmers. Therefore, an understanding of the reproductive biology of *P. viridis* is an indispensable aspect to develop sustainable farming technology and provide the plausible scientific advice for the management of this valuable resource.

An extensive number of studies have reported the gonadal development of *P. viridis* through macroscopic and histological characterization and revealed various stages of the reproductive cycle [6,7,8]. The identification of various gonad development stages by coloration is less reliable, particularly for the males, due to the contentious and overlapping coloration patterns of the gonads in some gametogenesis stages [9,10]. Male and female individuals of *P. viridis* are indifferent in their external morphological appearance [7]. Moreover, it is also nearly impossible to distinguish between males and females even from histological preparation during the spent and gonadal resting phases [6,7]. While the *P. viridis* is considered a dioecious species [11], evidence of hermaphroditism is also recorded in a very low percentage [12,13].

The reproduction of marine mollusks is a complex biological activity that is directly related to different exogenous (seasonality, environmental factors, food availability, etc.) and endogenous factors (stored energy, nutrient compositions, physiological conditions, etc.) [14,15,16]. However, a comprehensive study by integrating these factors and reproductive events has been rarely documented in mollusks, even for other aquatic species. Seasonality is the most dominating factor that influences the reproductive biology of mussels in both tropical and temperate countries [2,17]. Different environmental conditions may also result in different spawning times and duration, as well as more subtle variations in the gametogenic cycles of mussels [18]. In temperate countries, the spawning season of *P. viridis* is generally associated with the rising of seawater temperature, while in tropical countries, it spawns all year round [17,19]. Previous studies exposed that the spawning of mussels is largely influenced by temperature, salinity, and food availability [20,21]. Moreover, habitat was also found to influence the reproduction of mussels, where subtidal population had consistently higher gonad indices and prolonged reproductive maturity compared to the intertidal population [22].

Gametogenesis, a great energy and nutrient-consuming process, for mollusks intensively depends on stored energy reserves (conservative scheme) or uses energy derived directly from food (opportunistic scheme). Therefore, the nutrient supply to the gonads is crucial for the normal generation of gametes, which directly affects the reproduction. A previous study suggested that *P. viridis* adopts an opportunistic scheme to build up their gonads using energy from the available food [2]. As *P. viridis* is a suspension feeding bivalve [2], plankton is the main component of its diet, and an indispensable source of nutrients and energy for gametogenesis. However, the detailed effects of environmental parameters, food availability, and feeding behavior on the gametogenesis and spawning in *P. viridis* are still poorly understood.

The reproduction of marine bivalves depends on lipid components for metabolic energy and the generation of structural materials [23]. Therefore, high lipid content in the gonads was found to occur just prior to the spawning in many marine molluscan species [24]. Fatty acids are the major constituents of oocytes [25], and their accumulation in oocytes during gonad development is metabolized differently during the reproductive cycle. Some fatty acids such as polyunsaturated fatty acids (PUFAs) in the gonads are stored for structural purposes, while others are catabolized for energy [26]. As *P. viridis* adopts opportunistic schemes, variations of the gonadal fatty acid contents are closely related to the nature of ingested diets [27]. Marine phytoplankton are important food sources for *P. viridis* that contain a high amount of PUFAs [28]. However, the abundance of plankton is profoundly influenced by the seasonality and environmental factors [29]. *P. viridis* has the ability to selectively ingest plankton from the water column, depending on the environmental conditions and reproductive cycle [1,30]. Therefore, a rigorous study focusing on the interrelation among the environmental factors, feeding behavior, gonadal fatty acid contents, and gonad development is essential to obtain an extensive knowledge about the reproductive biology of *P. viridis*.

The objective of this study is to provide comprehensive knowledge about the reproductive biology of *P. viridis* in a multidisciplinary way including biometrics (body size, gender, and gonadosomatic index), morphological (gonad histology), biochemical (lipid content and fatty acid profile), and ecological (surrounding water quality parameters and feeding behavior) approaches during the annual spawning season. Moreover, the study applied a multivariate approach to the ecological and reproductive traits data to explore how the environmental factors, feeding behavior, and total lipids and fatty acids composition of the gonads of *P. viridis* are interrelated to the gametogenesis and maturation, emphasizing a pertinent effect of seasonality.

## 2. Materials and Methods

### 2.1. Sampling Strategies

Wild green mussels were sampled monthly in the period from November 2017 to October 2018 from the three locations, namely Choufaldandi (21°30′20′′ N and 91°59′19′′ E), North Khurushkul (21°27′54′′ N and 91°58′09′′ E), and Khurushkul (21°33′04′′ N and 92°03′14′′ E) of the Moheshkhali channel, Cox’s Bazar, Bangladesh. The channel is located on the southeastern coast of Bangladesh and directly connected to the Bay of Bengal. For this study, a total of 55–60 green mussels were randomly collected from the natural bed of these three collection sites by the local fishermen using diving equipment for each month. From these monthly collected samples of green mussels, about 20–22 green mussels were selected to identify the sex, gonadosomatic index, and gonadal histological study. Another 20 individuals of green mussels were selected to determine the monthly variation in ingested gut plankton content. The tentative gonad development stage of each of these individuals used for gut plankton analysis was also determined by observing the color and development mass of the gonad (please see Figure S1) to further calculate the quantity of gut plankton ingested according to different gonad development stages. From the remaining samples, 15 mussels were selected for gonad lipid content and fatty acid analysis for each month. After dissection and gonadal stage confirmation, the gonads of five individuals with same development stages were pooled together i.e., three pooled samples for each month from 15 individuals. Besides the monthly variation, these pooled samples during the annual spawning season were further grouped into different gonadal stages to present the gonadal lipid and fatty acid contents data in relation to the different gonad development stages. 

### 2.2. Biometric Measurements

For this purpose, a total of 242 green mussels were used throughout the annual reproductive cycle in which 20–22 green mussels were used for each month. Biometric measurements such as length (maximum length along the anterior–posterior axis), height (maximum length along the dorsal–ventral axis), and width (maximum length through both valves) of each individual were measured using a Vernier calliper with an accuracy of 0.01 mm. The total weight of each individual was recorded after draining the intervalval (or mantle) fluid to the nearest 0.1 g using an electronic balance (PS 1200.R2, Radwag, Poland). Both the male and female were dissected and the gonads were gently removed and weighed at 0.01 mg using the electronic balance (AS 220.R2, Radwag, Poland). The gonadosomatic index (GSI) was expressed as the percentage of the gonad weight in relation to the total body weight and calculated as:

$$\mathrm{GSI}\;=\;\frac{\mathrm{Weight}\;\mathrm{of}\;\mathrm{gonad}}{\mathrm{Total}\;\mathrm{weight}\;\mathrm{of}\;\mathrm{mussel}}\;\times \;100.$$

### 2.3. Histology of Gonad

After biometric measurement, the same 242 green mussels were used for histological analysis of the gonads throughout the annual reproductive cycle. For gonad histology, the gonadal masses were removed from the mantle lobes and fixed in Bouin’s solution for 24 h, and subsequently dehydrated in series (80–100%) of ethanol and xylene. Afterwards, the dehydrated gonads were embedded in paraplast, cut in transverse serial sections (7 µm), and mounted on slides. An increasing alcohol concentration protocol was adopted for dehydration of the mounted slide, and stained with Harrys’ hematoxylin and eosin [31]. The stained slides were observed and photographed using digital microscope (Optika B-190TB, Ponteranica, Italy) with magnification 10–40× for the identification of sex and gonad development stages. The condition of gonads was classified into five main categories including resting, development (stages A, B, C and D), mature, spawning (stage A, B and C), and spent [32]. 

### 2.4. Water Quality Assessments

For ecological factors analyses, 14 water quality parameters (collected from the same location of green mussel collection sites) were recorded monthly to investigate how these factors are related to the annual reproductive cycles and gametogenesis of *P. viridis.* A multifunction environmental sensor (YSI, Loveland, CO, USA) was used to measure in situ the temperature (°C), salinity (ppt), pH, and dissolved oxygen (DO, mg L^−1^). The water current (m sec^−1^) was measured with a digital water velocity meter (Flow Probe FP311, Global Water, College Station, TX, USA) positioned 0.5 m below the water surface. The Secchi depth transparency (m) and water depth (m) were also measured. For nutrients and chlorophyll-*a* determination, water samples were collected using a vertical water sampler (1200-E Kemmerar, Science First/WildCo, Yulee, FL, USA) and filtered through microfiber glass filter paper (Whatman GF/C), using a vacuum pressure air pump. Total alkalinity (titrimetric method) and NO_2_-N, NO_3_-N, NH_3_-N, NH_4_-N, and PO4-P concentrations were measured (PhotoFlex STD, WTW, Weilheim, Germany) on a monthly basis (APHA, 1992). Chlorophyll *a* was determined at 750-nm and 664-nm wavelengths (Optizen Pop 2102, Daejeon, Republic of Korea), following the previous method [33]. For water plankton measurements (cells L^−1^), 20 L of pooled water samples was passed through a 45-μm mesh plankton net, and the concentrated samples were preserved in small plastic bottles with 5% buffered formalin. A quantitative estimation of plankton was done using a Sedgewick–Rafter (S–R) cell containing 1000 1-mm^3^ cells [34]. A 1-mL sample was put in the S–R cell, and the plankton in 10 randomly selected cells were identified up to genus level and counted under a binocular microscope (Optika B-190TB, Ponteranica, Italy). Plankton abundance was calculated using the following formula: N = (P × C × 100)/L; where N is the number of plankton cells or units per liter of original water; P is the number of plankton counted in 10 fields; C is the volume of final concentrate of the sample (mL); and L is the volume (L) of the water sample.

### 2.5. Gut Plankton Analysis

The gut contents of 240 *P. viridis* (20 individuals per month) were examined throughout the annual reproductive cycle to ascertain how the ingested plankton contributed to the required nutrients and energy supply for gametogenesis. The length was standardized during the whole period of the study so that no variation in gut plankton contents could be a result of the sample being of different sizes. Immediately after collection, the major gonadal stages were tentatively determined by observing the color and development mass of the gonad (please see Figure S1). After collection and major gonadal stages determination, the mussels were preserved by using 20% buffered formalin for 7 days; subsequently, they were transferred to the 70% ethanol for 7 days followed by distilled water for another 4 days. Afterwards, the stomach content was collected by using a glass Pasteur pipette through a small slit beneath crystalline style and diluted in 50 mL of filtered sea water. A 1-mL subsample was transferred to a Sedgewick Rafter Counting Cell (S–R cell), and all plankton in 10 randomly selected squares were identified up to genus level and counted using a binocular microscope (Optica B-190TB with digital facilities; magnification 10×). For each gut sample, three subsamples were examined in a similar way. The abundance of the ingested gut plankton was calculated based on the formula: N = (P × C × 100), where N = numbers of plankton cells or units in the whole guts; P = total number of plankton counted in 10 fields; and C = volume of final concentrate of the sample in mL.

### 2.6. Lipid Content and Fatty Acid Analysis of Gonad

As mentioned previously, 15 mussels were collected on a monthly basis from November 2017 to October 2018, and the gonads of five individuals with the same development stages were pooled together i.e., three pooled samples for each month. Then, the pooled gonads were homogenized and freeze dried (FreeZone 4.5, LABCONCO, Kansas City, MO, USA) until constant weight and further divided into two portions. From one portion, lipids were extracted through a digital Soxhlet apparatus (FOOD ALYTRD40, OMNILAB, Bremen, Germany) with petroleum ether and acetone in a ratio of 2:1. The other portion (200–300 mg) was used for fatty acid determination by a one-step method [35]. The pooled gonad sample was esterified with 14% boron trifluoride (BF3) in methanol at 100 °C for 120 min, and hexane was used to dissolve the lipids. The fatty acid methyl esters (FAMEs) were separated and quantified by gas chromatography equipped with mass spectrometer (GC-2010 Plus, Shimadzu, Kyoto, Japan). The methyl ester of nonadecanoic acid (19:0, 99.6%, Dr. Ehrenstorfer GmbH, Augsburg, Germany) was used as an internal standard [36]. Qualitatively (as a percentage of total fatty acids), the composition of fatty acid was calculated by comparing the peak area of each fatty acid with the total peak area of all the fatty acids in the sample.

### 2.7. Statistical Analyses

All analyses were done using R, version 3.5.2 [37]. The assumptions of normal distributions of all datasets were checked with the Shapiro–Wilk test, and homogeneity of variances were checked with Levene’s test using the ‘onewaytests’ package [38]. The univariate analysis of variance (ANOVA) model was applied using the “car” package [39] followed by the Tukey multiple comparison test using the “multcomp” package [40] to compare the monthly variation of all the datasets. Significant differences were evaluated at the 95% confidence level. The correlations among variables were tested and plotted using the “PerformanceAnalytics” packages [41]. The principal component analysis (PCA) for all datasets was performed using the ‘FactoMineR’ package [42]. We used only the first and second PCs in all cases as they explained most of the variabilities. The PCAs and other plots were prepared using the ‘ggplot2’ package [43]. For the correlation analysis of ecological factors, we included the monthly GSI values together with water quality parameters. Besides correlation analysis, one-way ANOVA analysis was performed for the water quality dataset, which demonstrated that seasonality significantly (*p* < 0.001) influenced 12 out of the 14 water quality parameters except for alkalinity and NH_4_-N (Table S1). Therefore, a multivariate analysis by PCA was performed by using these 12 water quality parameters to know whether the multivariate spaces of monthly data corresponded to the gonad development cycle of *P. viridis*. We initially conducted correlation analysis between the GSI and the quantity of ingested gut plankton data. We included all the different groups of ingested gut plankton in PCA analysis, as the one-way ANOVA analysis showed that all the gut plankton parameters varied significantly (*p* < 0.008) with seasonality (Table S2) and different gonad development stages except Cyanophyceae (Table S3). The gonadal fatty acids data were expressed in percentages at first and then underwent an arcsine square root transformation before statistical analysis. Afterwards, we carried out the correlation test between the monthly data of major gonadal fatty acids (>1.0% of total fatty acids) and GSI values of *P. viridis*. Similarly, we conducted one-way ANOVA analysis for all 31 fatty acids and their groups, and demonstrated that the seasonality significantly influenced 21 fatty acids (Table S4), while the gonadal stage influenced 25 fatty acids (Table S5). Therefore, these 21 variables for a monthly variation dataset and 26 variables for a gonadal stage dataset were used for further PCA analysis. Finally, a correlation and PCA analysis were carried out using the 17 most important variables from the ecological factors, gut content, lipid content, and fatty acid data, which were found to be strongly correlated with the GSI value in this study, to better understand how these factors are interrelated among each other during the gametogenesis of *P. viridis*.

## 3. Results

### 3.1. Annual Reproductive Cycle

**Figure 1:**
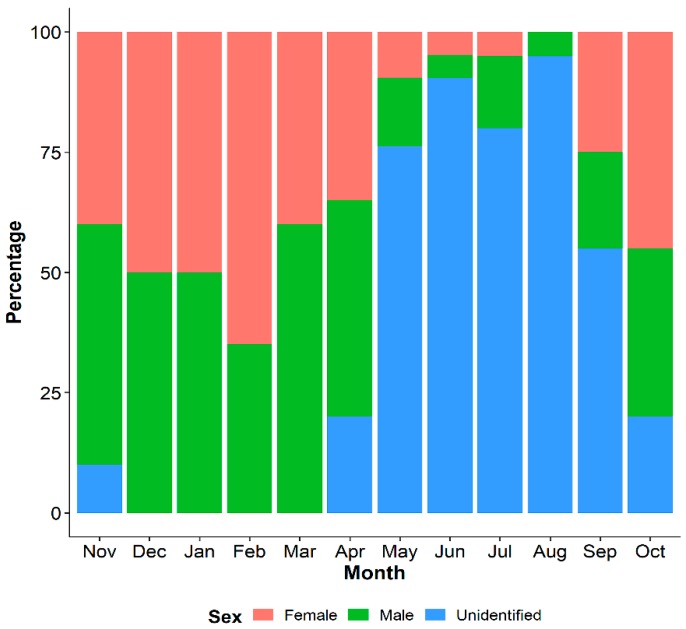
Proportion (%) of male, female, and undifferentiated individuals of *Perna viridis* collected from the coastal waters of Bangladesh.

The synchronous pattern of gonad development was observed for female and male individuals of *P. viridis*. The macroscopic observation showed that the intermantle and mesosomal gonads of female individuals had yellowish to light orange coloration in their developing stage, dark orange to red color in the mature stage, and a faded orange coloration with less reproductive cells during the spawning stage (Figure S1). The gonads of male individuals possessed a white to creamy white color, which was almost impossible to distinguish into different gonad development stages due to the overlapping color pattern. Moreover, we were unable to differentiate the sex of the majority of *P. viridis* individuals during May to September (gonadal resting phase) macroscopically by gonad coloration or even histologically (Figure 1). The histological analysis of 242 individuals precisely identified 67 (27.69%) as male and 62 (25.61%) as female, while 113 (46.69%) were undifferentiated. Overall, the sex ratio of males and females was found to be 1:0.93, which was not significantly deviated from the 1:1 ratio (χ2, *p* > 0.05). We did not find any hermaphrodite individuals during the study period.

**Figure 2:**
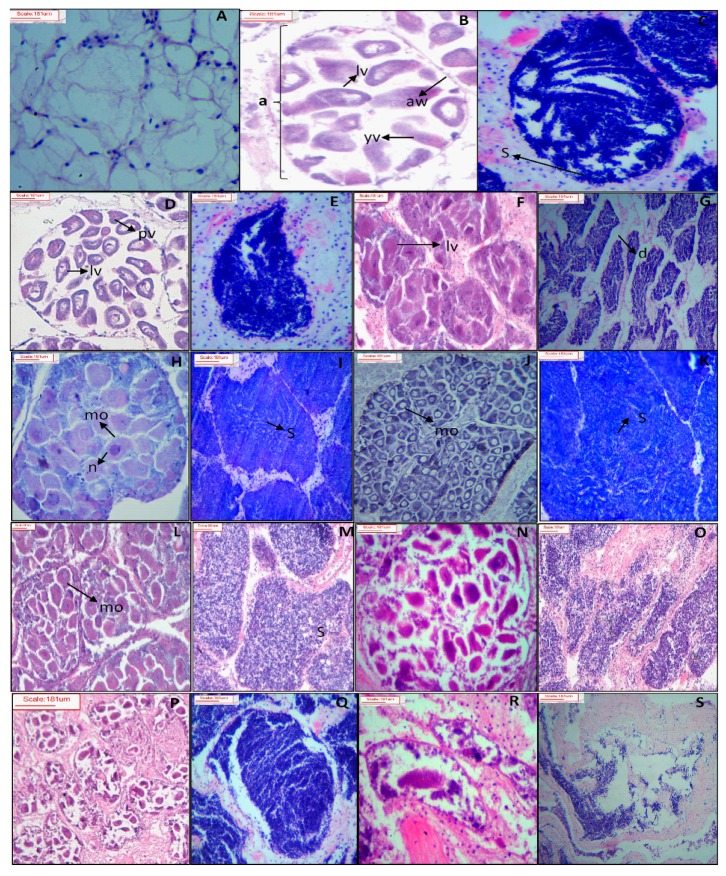
Transverse histological section of the female and male gonads of green mussel *Perna viridis* collected from the coastal waters of Bangladesh during November 2017 to October 2018. Resting stages (**A**), development stage A (**B**: female, **C**: male), development stage B (**D**: female, **E**: male), development stage C (**F**: female, **G**: male), development stage D (**H**: female, **I**: male), mature (**J**: female, **K**: male), spawning stage A (**L**: female, **M**: male), spawning stage B (**N**: female, **O**: male), spawning stage C (**P**: female, **Q**: male), and spent (**R**: female, **S**: male) are shown. Scale Bar: 181 µm. a, acinus wall; yv, young vitellogenic oocyte; lv, late vitellogenic oocyte; S, spermatozoa; pv, previtellogenic oocyte; lv, late vitellogenic oocyte; mo, mature oocyte; n, nucleus.

Five major gametogenesis stages of *P. viridis* were identified based on the oocyte and spermatocyte stages [32]. In the resting phase, males and females were undistinguishable, and the gonad consisted mostly of storage cells (Figure 2A). In the development stage, acini consisted of early vitellogenic, late vitellogenic, and mature oocytes in the female, while spermatophores had started to form in the male. This stage was further classified as development A (acinus just formed), B (30%), C (50%), and D (70%) based on the percentage of mature oocytes and spermatocytes in the gonad (Figure 2B–I). In the mature stage, the fully ripe mature ova of a polygonal shape were predominant with a reduction in developing oocytes in the female (Figure 2J), while ripe spermatozoa were dispersed densely with a very narrow duct space in the male (Figure 1K). During the spawning stage, gametes discharge was started and based on the gametes reduction percentage, this stage was further classified as spawning A (30%), B (50%), and C (70%) for both the male and female (Figure 2L–Q). Finally, at the spent stage, the acinus collapsed, and oocytes were reabsorbed in the female (Figure 2R), while sperms were broken down in the male (Figure 2S).

**Figure 3:**
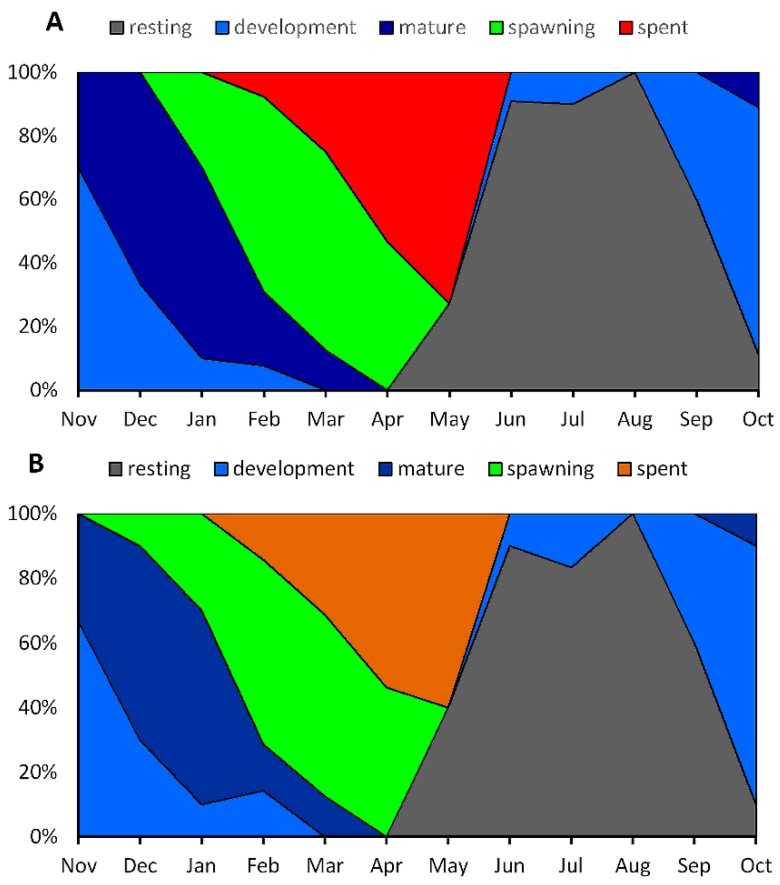
Monthly variations of gonadal maturity stages (%) in female (**A**) and male (**B**) *Perna viridis* collected from the coastal waters of Bangladesh during November 2017 to October 2018.

**Figure 4:**
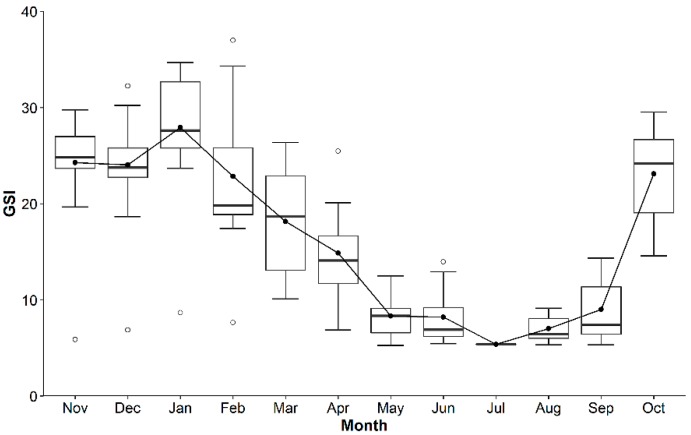
Box and whiskers plot of monthly variation of gonadosomatic index (GSI) of green mussel *Perna viridis* collected from the coastal water of Bangladesh during November 2017 to October 2018.

Histological analysis revealed that the active gametogenesis of *P. viridis* was observed annually in the coastal areas of Bangladesh (Figure 3A,B). Mass gonad development for both the male and female started from September after a long resting stage and reached the maturation stage early in November. In both sexes, rapid spawning occurred from January to April during late winter to early spring (Figure 3A,B). Undifferentiated gonads were evident from April in both sexes, indicating a resting phase before redevelopment (Figure 3A,B). The resting phase (May to August) continued until rapid gonad development in late autumn and early winter. The presence of spent gonads was evident from February to June in both the male and female *P. viridis*. However, a negligible percentage (<10%) of gonad development was also noticed in June and July. Seasonal variation in the gonadosomatic index (GSI) also reflected consistent results with the histological analysis of gonad development. An increasing shift of stipulating gonad development was observed as early as September, which reached toward its higher value in November to January, indicating the development and maturation stages of the gonads (Figure 4). After January, a decreasing inclination of GSI values from their peak until April indicated spawning stages of *P. viridis*, followed by the lower values in May to June representing the spent stage of the gonads (Figure 4). Moreover, the lowest GSI values during June to August indicated the resting stage of the gonads (Figure 4). Furthermore, the overall average value of the GSI of male and female individuals was found to be 23.6% and 22.4%, respectively with no significant (*p* > 0.05) difference between them.

### 3.2. Ecological Factors and Gametogenesis

**Figure 5:**
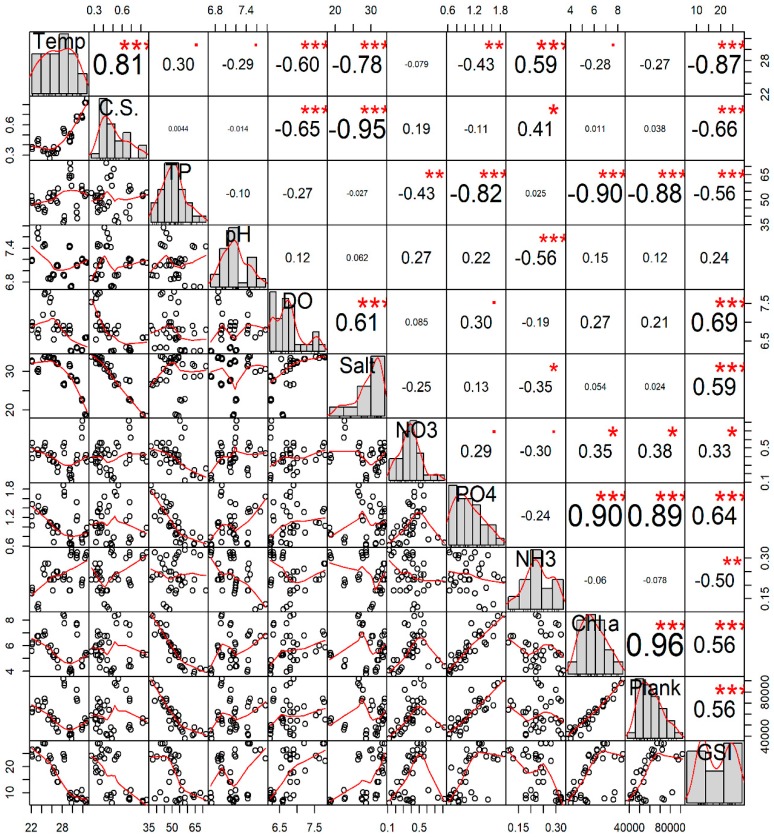
Interrelations among the different water quality parameters and the gonadosomatic index (GSI) of green mussel *Perna viridis* collected from the coastal waters of Bangladesh. The analysis is based on the three replicated measurements of water quality parameters for each month (n = 36). Here, the variables’ full names are: Temp, water temperature (°C); C.S., water current speed (m/s); TP, water transparency (cm); pH, water pH; DO, dissolved oxygen (ppm); Salt, salinity (ppt), NO3, nitrate-nitrogen (ppm); PO4, phosphate-phosphorus (ppm); NH3, ammonia-nitrogen (ppm); Chl.a, chlorophyll *a* (µg/L); Plank, water total plankton (cells/L); and GSI, gonadosomatic index (%). Values given around the all axis are the range of the individual parameters’ measured values. Correlation coefficients are indicated with numeric values, while significance levels are denoted by asterisks (* < 0.05, ** < 0.01, *** < 0.001).

The results revealed that GSI has a significant positive correlation with DO (r = 0.69; *p* < 0.001), salinity (r = 0.59; *p* < 0.001), NO_3_–N (r = 0.33; *p* < 0.05), PO_4_–P (r = 0.64; *p* < 0.001), Chlo-a (r = 0.56; *p* < 0.001), and water plankton abundance (r = 0.56; *p* < 0.001), and significant negative correlation with temperature (r = −0.87; *p* < 0.001), current speed (r = −0.66; *p* < 0.001), transparency (r = −0.56; *p* < 0.001), and NH_3_–N (r = −0.50; *p* < 0.01) (Figure 5). Other water quality parameters such as water depth (r = 0.001), pH (r = 0.24), alkalinity (r = 0.013), NO_2_–N (r = 0.11), and NH_4_-N (r = 0.15) did not show any significant correlation (*p* > 0.05) with the GSI values of *P. viridis*.

**Figure 6:**
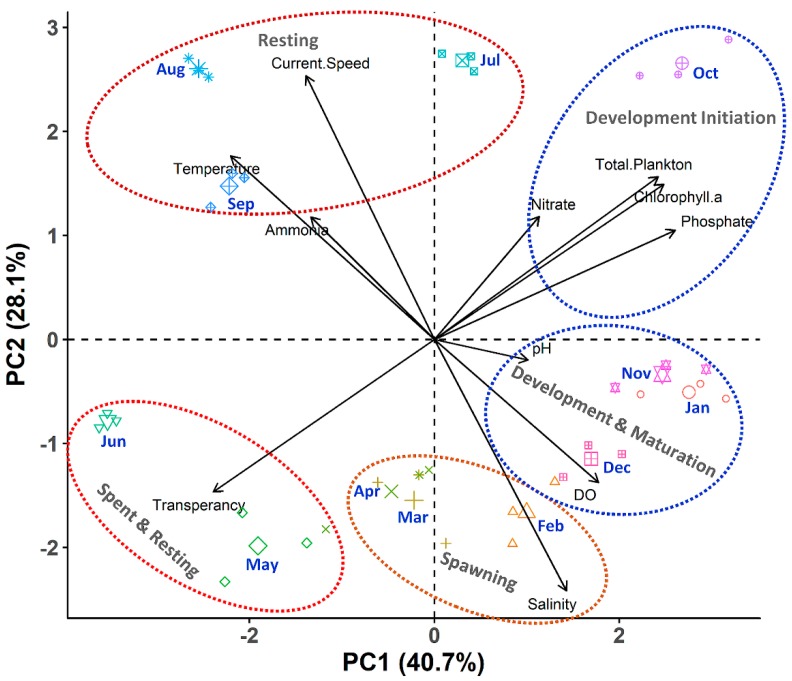
Biplot of principal component analysis (PCA) of the monthly variation of different water quality parameters of the Moheshkhali channel of Bangladesh (green mussel collected sites) for the period of November 2017 to October 2018. The analysis is based on the three replicated measurements of water quality parameters for each month (n = 36).

The PCA analysis revealed that the first three principal components explained 82.83% of the variation, having eigenvalues >1 (Table S6). The monthly variation data of water quality parameters were separated into multivariate spaces corresponding to the different gonadal stages of *P. viridis* (Figure 6). It was observed from PC1 that phosphate, chlorophyll *a*, and total plankton largely contributed to the developmental stage during the month of October, while salinity and DO had major contributions to the maturation and spawning stages during the months from November to February (Figure 6 and Table S6). It was revealed from PC2 that the current speed and temperature had major contributions to the resting stage during the months of August and September, while transparency was the major single factor, which was related to the spent and resting stages during the months from April to June (Figure 6).

### 3.3. Feeding Behavior and Gametogenesis

**Figure 7:**
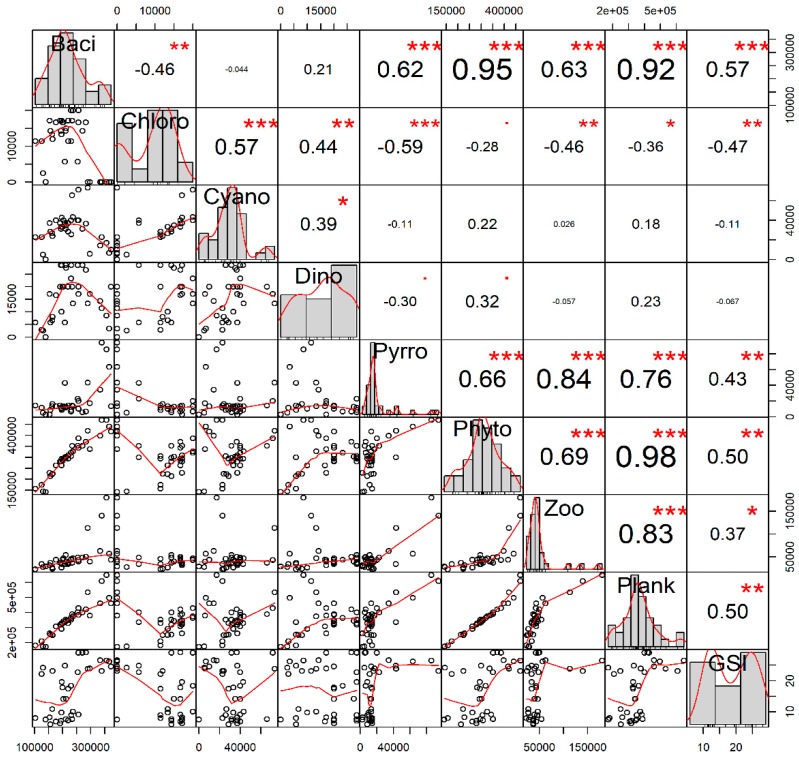
Interrelations among the major groups of the ingested gut plankton (quantity of individuals in whole gut) and the gonadosomatic index (%) of green mussel *Perna viridis* collected from the coastal waters of Bangladesh during November 2017 to October 2018. The analysis is based on the 20 individuals of green mussel for each month (n = 240). Here, the variables’ full names are: Baci, Bacillariophyceae; Chloro, Chlorophyceae; Cyano, Cyanophyceae; Dino, Dinophyceae; Pyrro, Pyrrophyceae; Phyto, total phytoplankton; Zoo, total zooplankton; Plank, total plankton; and GSI, gonadosomatic index. The values given around all the axes are the range of each individual parameter’s measured values. Correlation coefficients are indicated with numeric values, while significance levels are denoted by asterisks (* < 0.05, ** < 0.01, *** < 0.001).

During the study period, a total of 51 phytoplankton species under the groups of Bacillariophyceae (thirty three), Chlorophyceae (three), Cyanophyceae (three), Dinophyceae (eight), Pyrrophyceae (four), and 15 zooplankton species were recorded in the gut contents of *P. viridis* (data not shown). Interestingly, correlation analysis revealed that the quantity of ingested Bacillariophyceae (r = 0.57, *p* < 0.001), Pyrrophyceae (r = 0.43, *p* < 0.01), total phytoplankton (r = 0.50, *p* < 0.01), zooplankton (r = 0.37, *p* < 0.05) and total plankton (r = 0.50, *p* < 0.01) showed significant positive correlation, while only Chlorophyceae (r = −0.47, *p* < 0.01) had shown significant negative correlation with the GSI value of *P. viridis* (Figure 7). However, the ingested Cyanophyceae (r = −0.11, *p* > 0.05) and Dinophyceae (r = −0.067, *p* > 0.05) had no significant correlation with the GSI value of *P. viridis* (Figure 7).

**Figure 8:**
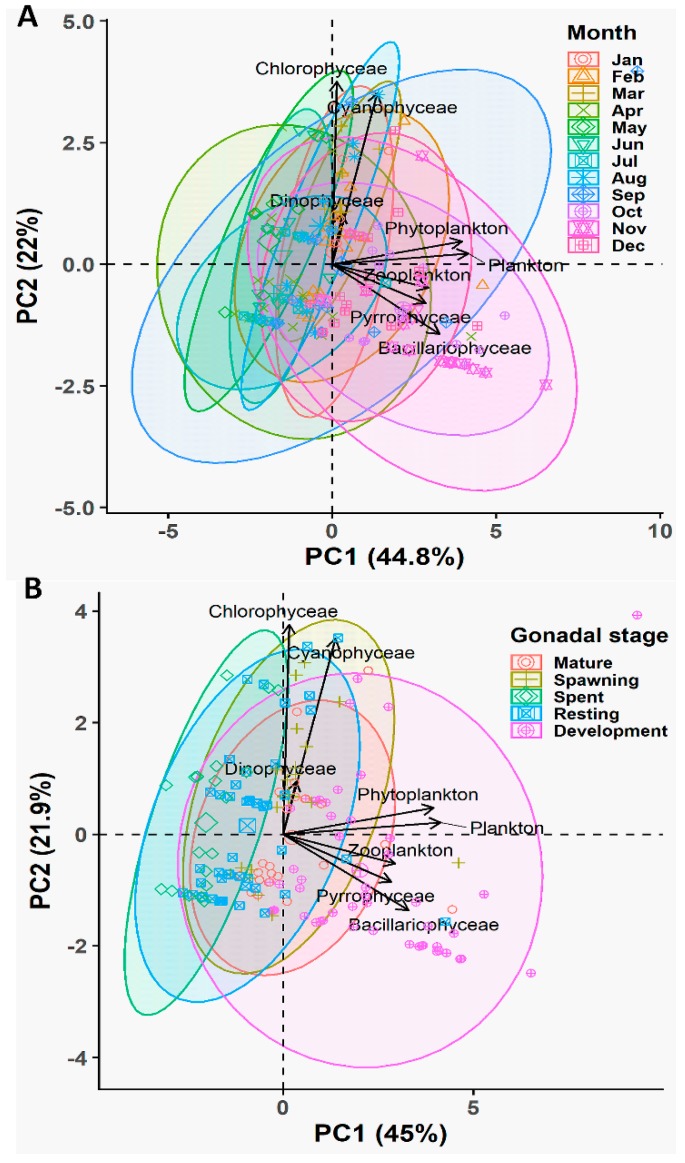
Biplot of principal component analysis (PCA) of the major groups of the ingested gut plankton at different months (**A**) and different gonadal stages (**B**) of green mussel *Perna viridis* collected from the coastal waters of Bangladesh during November 2017 to October 2018. The PCA of the monthly variation of the gut content analysis consisted of the 20 replicated green mussels for each month (n = 240), whereas the PCA of the different gonadal stages consisted of 37 mature, 34 spawning, 30 spent, 79 resting, and 60 developing stage green mussels (n = 240).

The ANOVA results demonstrated that *P. viridis* ingested the highest amount of plankton, particularly Bacillariophyceae (*p* < 0.001), during their development stage, followed by the mature and spawning stages, while the lowest ingestion was observed during the spent and resting stages (Table S3). We further performed multivariate analysis using PCA to reduce the dimensionality of the obtained information (Figure 8A,B). We extracted three principal components (PCs) that accounted for 79.9% of the variability for the seasonal variation dataset (Table S7) and 80.25% of the variability for the gonadal stage dataset (Table S8). In both datasets, PCA biplots displayed that the multivariate spaces exhibited very overlapping patterns among different months as well as different gametogenesis stages (Figure 8A,B). However, the multivariate spaces of development and mature stages of gametogenesis, particularly during October and November, are well separated and distinguishable. Furthermore, PCA analysis also revealed that Bacillariophyceae, Pyrrophyceae, total phytoplankton, and total zooplankton contributed largely to PCI, while the Chlorophyceae and Cyanophyceae had major contributions to PC2 in both datasets. Based on the all analyses, it was evident that *P. viridis* ingested a higher amount of phytoplankton, particularly Bacillariophyceae and Pyrrophyceae during the months of October and December, which corresponds to their gonad development and maturation stages.

### 3.4. Gonadal Lipid and Fatty Acid Profiles

**Figure 9:**
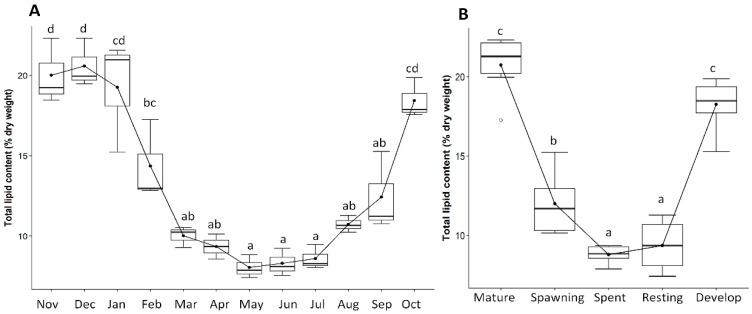
Box and whiskers plots of the variation of gonad lipid contents (% dry weight) of green mussel *Pirna viridis* during different months (**A**) and gonadal stages (**B**). Superscript letters indicate significant differences (*p* < 0.05).

**Figure 10:**
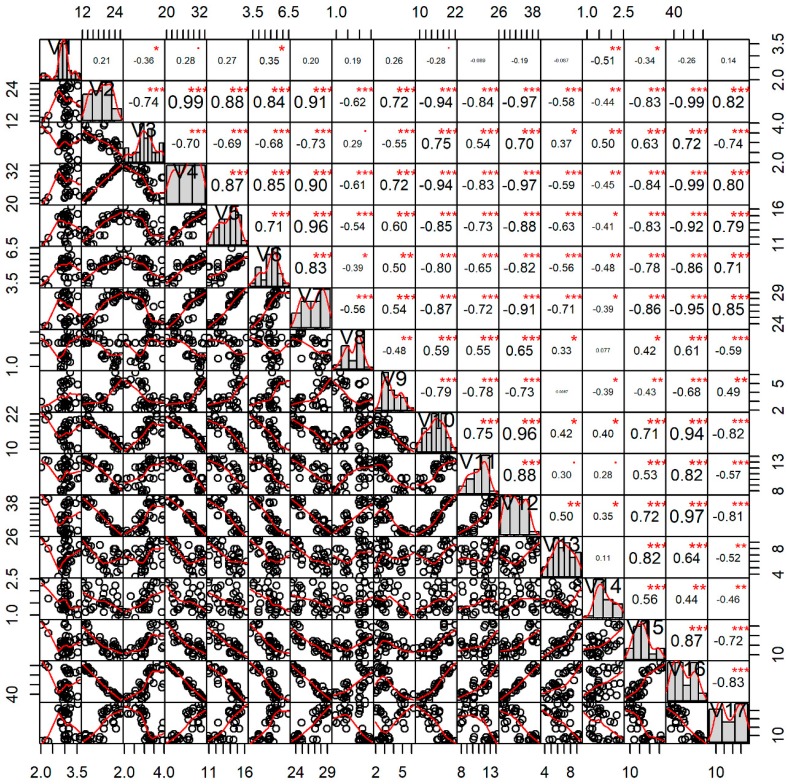
Interrelation among the major gonadal fatty acids (fatty acids >1.0%) and the gonadosomatic index (GSI) of green mussel *Perna viridis* collected from the coastal waters of Bangladesh. The correlation analysis is based on the three replicated pooled samples (each pooled sample consisted of gonads from five green mussels) for each month (n = 36). Here, the variables’ full names are: V1, C14:0; V2, C16:0; V3, C18:0; V4, total SFAs; V5, C16:1; V6, C18:1n-9; V7, total MUFAs; V8, C18:3n-3; V9, C18:4n-3; V10, C20:5n-3; V11, C22:6n-3; V12, total n-3 polyunsaturated fatty acids (PUFAs); V13, C20:4n-6; V14, C22:2n-6; V15, total n-6 PUFAs; V16, total PUFAs; and V17, gonadosomatic index (%). Values given around all the axes are the range of each individual fatty acid/group’s measured value as the percentage of total fatty acids. Correlation coefficients are indicated with numeric values, while significance levels are denoted by asterisks (* < 0.05, ** < 0.01, *** < 0.001).

The information of gonadal lipid contents showed significant effects of seasonality (Figure 9A) and varied significantly among different gametogenesis stages (Figure 9B). It started to increase during the late resting stage (August to September), reached its maximum during the development and mature stages (October to December), and afterwards tended to decrease during the spawning season (January to April), and finally downwards to its minimum value during the spent and resting stages (May to July) (Figure 9A,B). Correlation analysis revealed that most of the fatty acids were strongly correlated with the GSI value except for the myristic (C14:0) and arachidonic (C20:4n-6) acids (Figure 10). Among the significantly correlated major fatty acids, C16:0 (r = 0.70, p < 0.001), saturated fatty acids (SFAs) (r = 0.68, *p* < 0.001), C20:5n-3 (r = 0.74, *p* < 0.001), C22:6n-3 (r = 0.71, *p* < 0.001), n-3 PUFAs (r = 0.77, *p* < 0.001), and PUFAs (r = 0.73, r = 0.71, *p* < 0.001) were positively correlated, while C16:1 (r = −0.83, *p* < 0.001), C17:1 (r = −0.59, *p* < 0.001), C18:1n-7 (r = −0.69, *p* < 0.001), C20:1n-9 (r = −0.60, *p* < 0.001), monounsaturated fatty acids (MUFAs) (r = −0.84, *p* < 0.001), C18:4n-3 (r = −0.66, *p* < 0.001), and n-6 PUFAs (r = −0.48, *p* < 0.01) were negatively correlated with the GSI value of *P. viridis*.

**Figure 11:**
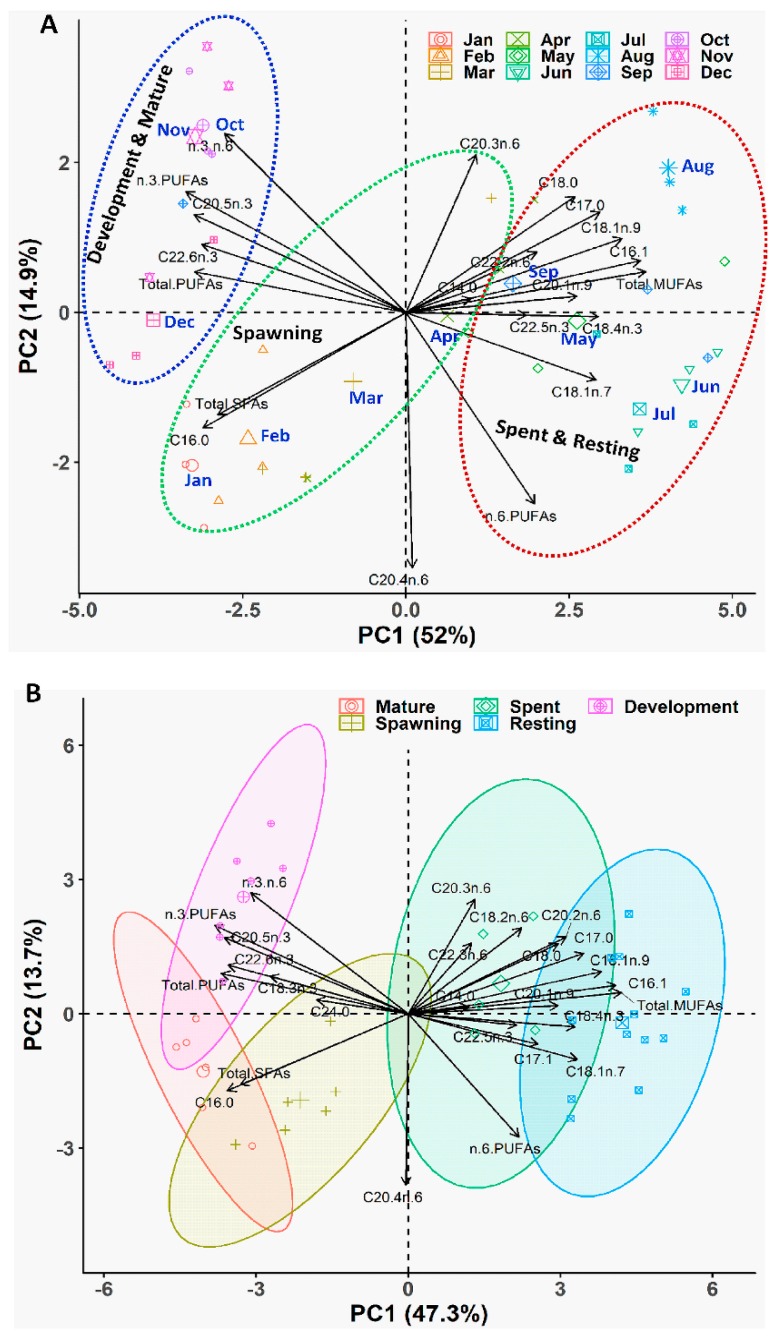
Biplot of principal component analysis (PCA) of the fatty acids at different months (**A**) and different gonadal stages (**B**) of green mussel *Perna viridis* collected from the coastal waters of Bangladesh. The PCA analysis is based on the three replicated pooled samples (each pooled sample consisted of five of the same gonadal stage green mussel) for each month (n = 36), whereas the PCA analysis of different gonadal stages consisted of six mature, six spawning, five spent, 12 resting, and seven development stage-specific pooled samples (n = 36).

Applying the PCA, we extracted five PCs that accounted for 86.66% of the variability for the seasonal variation dataset (Table S9) and 79.62% of the variability for the gonadal stage dataset (Table S10). Biplots of the PCA displayed that multivariate spaces corresponded to the gametogenic cycle of *P. viridis* (Figure 11A,B). In both datasets, C17:0, C18:1n-9, C18:1n-7, total MUFAs, and C18:4n-3 were the major contributing fatty acids in PC1, which are mainly related to the spent and resting stages (Figure 11A,B). C18:0, n-3 PUFAs, C20:2n-6, and n-3:n-6 were accounted for as major contributors to PC2, which were related to the development and mature stages. In both PCs, C16:0 and total SFAs had large negative loadings, which were responsible for the spawning period of this species (Figure 11A,B).

The outcomes of all analyses demonstrated that gonads accumulated a high amount of SFAs during maturation and spawning (January to April), MUFAs during the spent and resting stages (May to September), and PUFAs during the development and maturation stages (October to December). Precisely, n-3 PUFAs were found to have higher percentages during the development stages, while n-6 PUFAs were found to have higher percentages in the spawning and resting stages (Figure 11). PCA biplot and ANOVA results also revealed that among SFAs, higher percentages of C14:0, C17:0, C18:0, and C20:0 were accumulated in gonads during the spent and resting stages, while C16:0 and C24:0 were accumulated more during maturation and the spawning season. Among the n-3 PUFAs, higher percentages of C18:3n-3, C20:5n-3, and C22:6n-3 were accumulated during the development and maturation stages, while C18:4n-3 and C22:5n-3 were accumulated in the spent and resting phases. Consistently, the higher ratio of n-3:n-6 PUFAs were also found during the development and maturation stages from October to December.

### 3.5. Interrelations among Different Factors

**Figure 12:**
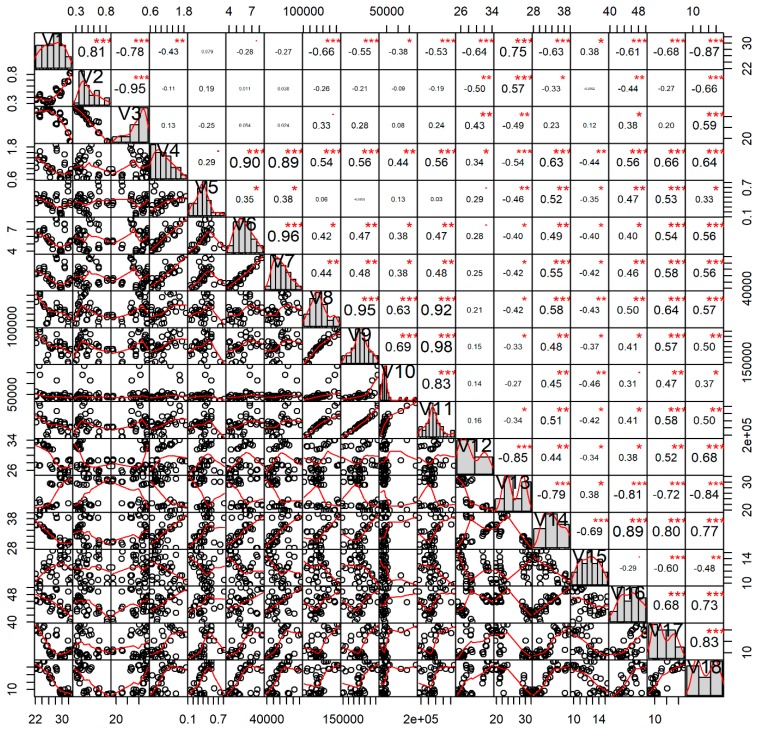
Interrelations among various water quality parameters, gonadal fatty acids, ingested gut planktons and gonadosomatic index (GSI) values of green mussel *Perna viridis* collected from the coastal waters of Bangladesh for the period from November 2017 to October 2018. Here, the variables’ full names are: V1, water temperature (°C); V2, water current speed (m/s), V3, water salinity (ppt); V4, water phosphate-phosphorus (ppm); V5, water nitrate-nitrogen (ppm); V6, water chlorophyll *a* (µg/L); V7 water total plankton (cells/L); V8, gut Bacillariophyceae (individuals in whole gut); V9, gut total phytoplankton (individuals in whole gut); V10, gut total zooplankton (individuals in whole gut); V11, gut total plankton (individuals in whole gut); V12, gonad SFAs (% of total fatty acids); V13, gonad MUFAs (% of total fatty acids); V14, gonad n-3 PUFAs (% of total fatty acids); V15, gonad n-6 PUFAs (% of total fatty acids); V16, gonad total PUFAs (% of total fatty acids); V17, gonad total lipid (% dry weight); and V18, gonadosomatic index (%). Values given around all the axes are the range of each individual parameter’s measured values. Correlation coefficients are indicated with numeric values, while significance levels are denoted by asterisks (* < 0.05; ** < 0.01; *** < 0.001).

The outcomes of correlation analysis represented complex interrelationships among different parameters of ecological factors, gut plankton contents, gonad lipid and fatty acids profile, and GSI values of *P. viridis* (Figure 12). Primarily, it was observed that increased nutrients and chlorophyll-*a* concentration was positively correlated with the high plankton availability in the water column. Such high availability of the plankton facilitated the higher ingestions of different groups of plankton by *P. viridis.* Moreover, temperature and other factors were also found to influence the plankton ingestion rate in this species from the water column. Interestingly, it was observed that n-3 PUFA accumulation in the gonad was positively correlated with the amount of ingested plankton (r = 0.51; *p* < 0.01), particularly Bacillariophyceae (r = 0.58; *p* < 0.001). In contrast, the MUFA (r = −0.84; *p* < 0.001) and n-6 PUFA (r = −0.48; *p* < 0.01) content in the gonads displayed negative correlation with the amount of ingested gut plankton. Lastly, lipid content and the GSI value of the gonads were influenced by the environmental factors, plankton availability, amount and types of the ingested plankton, and fatty acid content of the gonads (Figure 12).

**Figure 13:**
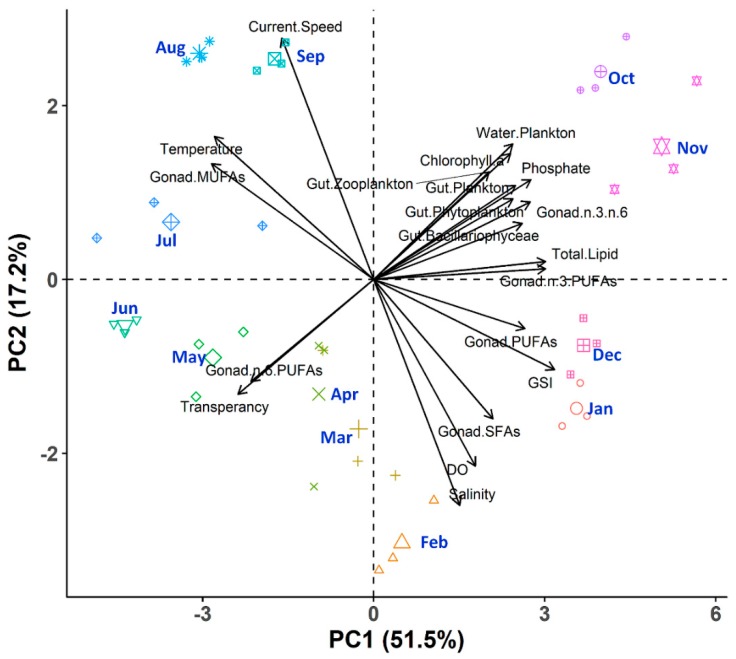
Biplot of principal component analysis (PCA) showing the relationships among the environmental factors, ingested gut plankton, lipids, and fatty acid content of gonads and GSI of green mussel *Perna viridis* collected from the coastal waters of Bangladesh for the period of November 2017 to October 2018. The analysis consists of 36 observations and the 20 most important variables from the environmental factors, ingested gut plankton, lipids, and fatty acid content of gonads and GSI.

A multivariate PCA analysis was also performed to provide further extensive knowledge about the interrelationship of the multiplex scenario with seasonality that summarized the information contained from the previously described datasets (Figure 13). Applying PCA to this dataset, we extracted three PCs that explained 79.22% of the variability in the original data (Table S11). The first PC (PC1) accounted for 51.47% of the variability and was dominated by GSI, the second PC (PC2) explained 17.17% of the variability and was mainly represented by current speed, and the third PC considered 10.58% of the variability and was dominated by ingested gut planktons. As the third component explained only 10.58% of the variance, it was not considered for further steps. The PCA outcomes was consistent with the correlation analysis and provided an additional opportunity to interpret the outcomes in terms of seasonality (Figure 13). During October to November, nutrient concentration, plankton abundance, plankton ingestion, total lipids, n-3 PUFAs, and n-3: n-6 PUFAs were positively interrelated among each other and triggered the gonad development and maturation of *P. viridis*. During December to March (maturation and spawning), GSI, DO, salinity, SFAs, and PUFAs were strongly correlated among each other. Whereas, during May to September, gametogenesis followed the spent and resting stages, and was characterized by the positive interrelation among the temperature, current speed, transparency, MUFAs, and n-6 PUFAs.

## 4. Discussion

Studies revealed that the interactions among various ecological and physiological factors largely control the duration and timing of the reproductive cycle of marine bivalves [15,16]. Therefore, the present study was designed to apply a multidisciplinary approach to explore a deeper understanding about how the ecological factors, feeding behaviors, gonadal lipids, and fatty acid levels are interrelated among each other to control the reproductive cycle of green mussels, highlighting a pertinent effect of seasonality.

The gametogenesis of *P. viridis* was accompanied with changes in gonad color for both male and females. However, our findings suggested that a macroscopic morphological identification of gametogenesis stages is not reliable based on only the gonad coloration for this species [7]. Although 46.69% of the collected specimens were unidentified for sex differentiation due to a long resting phase, our results showed that the green mussel is a dioecious species and the sex ratio was not significantly deviated from 1:1, which corroborates other studies [7,11]. GSI is a well-known index of the reproductive condition of an animal—an increase in its value indicates gametogenesis, while a decrease from its peak denotes spawning. As a broadcast spawner, the male GSI was found to be relatively higher than that in the female to ensure success in fertilization during spawning [10]. Depending on the geographical location and environmental conditions, *P. viridis* was reported to spawn year round [44], twice a year [17], or only once in a year [45]. In the coastal waters of Bangladesh, *P. viridis* followed an annual pattern of the reproductive cycle with a single spawning event, while two peak spawning activities were also reported along the neighboring country in the Bay of Bengal region [17]. The expulsion of gametes usually occurred annually from February to April during the late winter and early spring seasons, indicating that the temperature threshold did not induce spawning in *P. viridis* but rather by warming from the winter [46]. After spawning, a long resting phase during the summer and monsoon was present as the temperature rises to its maximum, but salinity and food availability decreased due to heavy rainfall and freshwater runoff [47]. Gonad development started in October and was associated with the increased availability of nutrients and plankton in the waters.

The reproduction of marine bivalves is primarily driven by the surrounding ecological factors and food availability [6]. In our study, there was a clear signal of seasonal variability of many ecological factors, most of which also had a strong correlation with GSI value. Therefore, seasonality explained a great deal of the variability in the gonad development of green mussels. More specifically, we observed that the seasonal variation of salinity and plankton abundance was positively correlated with the GSI values, which indicates that these factors may positively influence the gametogenesis of *P. viridis*. The PCA analysis further confirmed that food availability had large positive loadings, particularly during the onset of gonad development stages at the month of October, while the maturation and spawning stages of *P. viridis* were associated with higher salinity during November to April (see Figure 6). Previous studies on the gametogenic cycle of *P. viridis* indicated that the temperature, salinity, and food availability fundamentally influenced the gonad development and spawning [17,48]. Although *P. viridis* is known to tolerate wide ranges of salinity (5.2 to 39.8 ppt), the prevalent salinity level (27 to 34 ppt) during the months of October to April was found to be within the optimum range for reproduction and growth [1,7]. During those periods, enhanced levels of phosphate and nitrates augmented the growth of plankton as revealed by a strong positive correlation between them in this study (see Figure 12). Moreover, those nutrient concentrations also had a significant positive correlation with chlorophyll*-a* concentration, further confirming the increased abundance of phytoplankton due to the high availability of these nutrients. As *P. viridis* is a suspension feeding bivalves, the higher abundance of plankton stimulated the gametogenesis by supplying adequate nutrients and the energy required for it [2]. It was also reported that the gametogenesis of *P. viridis* is not only influenced by phytoplankton abundance, but also by its composition; rapid gonadal development was observed in the presence of a high density of *Coscinodiscus* spp. [2]. Interestingly, we also observed an increased abundance of *Coscinodiscus* spp. during those periods (October to December) in the coastal areas of Bangladesh (data not given). In contrast, the temperature, current speed, and transparency were negatively correlated with GSI values, indicating that the higher values of those parameters may have stalled the onset of gametogenesis. Consistently, the PCA analysis also confirmed that the resting stages of gametogenesis during May to August were associated with a higher temperature, current speed, and transparency (see Figure 6). During the summer and monsoon seasons (May to September), the coastal waters of Bangladesh had very high temperatures together with a faster current speed, low salinity, depleted nutrient concentration, and lower availability of plankton due to heavy rainfall and surface runoff. Although *P. viridis* showed a positive shift to tolerate higher temperatures [49], the rising temperature had a proven negative impact on reproduction in mussels, as a low concentration of nutrients was observed in the Gulf of Aden due to this high temperature [50]. Extremes of temperature and desiccation lead to the gametes’ degeneration and resorption in mollusks [51]. The observed high current speed (≥0.5 ms^−1^) during those periods was far above the recommended optimum levels of 0.1 to 0.3 ms^−1^ for growth and reproduction [1,52]. High current speed was reported to cause disturbances to mussel settlement and furthermore breeding [53,54]. Therefore, nonoptimal values of these ecological factors either individually or simultaneously retarded the onset of gametogenesis or prolonged the resting stage of *P. viridis* from May to September [47].

The marine bivalves can select specific food elements to meet the special and unique metabolic requirements of crucial stages during gametogenesis [2,30]. In our study, gut content analysis demonstrated that varieties of phytoplankton (51 genus) represented the most important food constituents for *P. viridis* [55,56]. Besides phytoplankton, our study also revealed that a large number of zooplankton (15 genus) served as an important source of additional food items [57,58]. Previous studies reported that *P. viridis* selectively ingested plankton from the water column depending on the environmental conditions and reproductive cycle [1,30]. Consistently, we also observed that the GSI value of *P. viridis* was positively correlated with Bacillariophyceae, Pyrrophyceae, and zooplankton, whereas it was negatively correlated with Chlorophyceae indicating the preferential selective feeding on certain groups of plankton by *P. viridis* during gametogenesis. This kind of selective feeding behavior was also reported for many other marine bivalve species [59,60,61]. The positive correlation between selective ingestion and GSI values further supports the concept of feeding behavior in bivalve that is related to the nutritional demands during gametogenesis [30]. Consistent with the correlation analysis, the PCA analysis revealed that the ingestion of different groups of plankton displayed a very overlapping pattern in different months except for October to December, particularly during the gonad development and maturation stages (see Figure 8). As gametogenesis is an energy-demanding process, the ingestion of a higher quantity of plankton during the gonad development stages further confirmed that *P. viridis* adopted opportunistic strategies to build up their gonads using energy from the available food [1] similar to other tropical bivalve species [60,61]. Among the different plankton groups, the observed large positive loadings of Bacillariophyceae, Pyrrophyceae, and zooplankton in PCA analysis during the gonad development stage further indicated the distinctive metabolic requirements of the specific nutrients by *P. viridis* during gametogenesis [30]. It was reported that Bacillariophyceae is an important food source for marine bivalves due to its high content of polyunsaturated and unsaturated fatty acids [28]. Although the data of genus-wise ingestion was not presented in this study, quantitatively, *Coscinodiscus* spp. alone in the stomach content of *P. viridis* accounted for more than 50% of the total Bacillariophyceae and 35% of the total plankton, indicating that the selective ingestion of this species could partly be related to this energy-demanding physiological process [30]. For marine bivalves, *Coscinodiscus* spp. are well-known nutritive phytoplankton taxa [62].

In marine bivalves, studies revealed that lipids play a crucial role for supplying metabolic energy and structural materials during the reproduction process for the formation of cell membranes [63,64]. In general, lipid accumulation in the body and gonads of many animals increased as the gametogenesis stages progressed toward spawning. A strong correlation between the phase of the reproductive cycle and the lipid content of the gonads was revealed in several marine bivalve species [64,65]. In agreement with those findings, our study also unveiled a strong positive correlation between GSI value and gonadal lipid contents (see Figure 10). Furthermore, the observed higher lipid content in the gonads during the development and maturation stages (October to December) just before spawning further confirmed that *P. viridis,* as in other marine bivalves, accumulated lipid globules in the gametes as an energy source for reproductive activity [24,66]. The gonadal lipid content gradually decreased when gametes were released for fertilization and finally reached its lowest level in the spent stage, because they are metabolized and being lost during spawning.

Fatty acids also provide energy during gametogenesis in marine animals; spermatozoa needed them for swimming during fertilization [67], while they are crucial for larval development and survival in ova [68]. The fatty acid analysis of the *P. viridis* gonads revealed that palmitic acid (C16:0) from SFAs, palmitoleic acid (C16:1) from MUFAs, eicosapentanoic acid (EPA) (C20:5n-3) and docosahexaenoic acid (DHA) (C22:6n-3) from n-3 PUFA, and arachidonic acid (C20:4n-6) from n-6 PUFA were the highest contributing fatty acids associated with the gonad development. Thus, the present study indicated that the gonadal fatty acid contents in *P. viridis* during gametogenesis conforms to the common trend, which is the characteristic of marine bivalves, other molluscs, and marine animals in general. The principal SFAs, MUFAs, and PUFAs in gonads were generally similar to those in most marine bivalve species during gametogenesis, although the abundance of each fatty acid in different bivalves might vary considerably. In the present study, a clear distinction of fatty acids accumulation in the gonads of *P. viridis* was observed among different gametogenesis stages at different seasons (see Figure 11). The gonads of *P. viridis* accumulated high amounts of SFAs during spawning (January to April) probably for use as an energy source during embryogenesis and the early larval stages [69]. Furthermore, they accumulated a high quantity of PUFAs during the development and maturation stages (October to December) possibly for utilizing them during embryogenesis as an energy source and as a constituent of the cell membranes [70]. In contrast, the MUFAs content was found to be at a lower level during the advanced gametogenesis stages, plausibly indicating that some of the MUFAs might be converted into PUFAs during the active requirement stages of gametogenesis [70]. Moreover, we also observed a strong negative correlation (r = −0.81) between MUFAs and PUFAs, further supporting the concept of biosynthesis of PUFAs. Although little is known about fatty acids biosynthesis in *P. viridis*, the biochemical studies revealed that marine bivalves have some ability to synthesize the PUFAs in the gonads, particularly at more advanced stages of development [71,72]. However, such capability appears to vary among species depending upon on the enzymatic complement of desaturase and elongase enzymes involved in these metabolic reactions [70].

Besides the gametogenic cycle, studies revealed that the fatty acids content of the gonads in marine bivalves are also influenced by the environmental factors affecting metabolism, the quantity and quality of available nutrients, and feeding behavior [73,74]. In the present study, the correlation analysis revealed a strong relationship of different groups of fatty acids with the surrounding environmental factors, particularly temperature, salinity, and current speeds (see Figure 12). A previous study also reported that the total content of PUFAs was differed predominantly due to the combined effect of the temperature and salinity of the habitat [75]. The observed positive correlation between PUFAs content and the quantity of ingested planktons in this study further suggested that a variation in fatty acids contents during different gonad development stages was closely related to the fatty acid contents of planktons [27]. The high content of the gonadal PUFAs during the advanced gametogenesis stages of *P. viridis* was due to the increased ingestion of plankton, particularly Bacillariophyceae and dinoflagellates, which contain higher amounts of n-3 fatty acids [76,77]. More precisely, it was reported that Bacillariophyceae are rich in eicosapentanoic acid (EPA), while dinoflagellates (Pyrrophyceae) are enriched with docosahexaenoic acid (DHA) [76,77]. In our study, it was revealed that during the gonadal mature stages, *P. viridis* consumed more than two times the amount of Bacillariophyce and Pyrrophyceae compared to that during the spent and resting stages (see Table S3). Nevertheless, all the nutrients proceeding from the different groups of phytoplankton and zooplankton described in the study were utilized by *P. viridis* to maintain the gametogenesis process.

## 5. Conclusions

In the present study, we used a multidisciplinary approach to provide an extensive knowledge about the reproductive biology of *P. viridis*. The integrated approach revealed that *P. viridis* has an annual reproductive cycle that is strongly interlinked and influenced by the seasonal variation of a set of environmental factors, feeding behaviors, and gonadal lipid and fatty acid contents. During the post monsoon season (October to January), gonad development and maturation were triggered by a set of environmental conditions, such as reduced temperature and current speed along with the enhanced concentration of salinity, DO, nutrients, and planktons. At this season, *P. viridis* selectively ingest a higher amount of planktons, particularly Bacillariophyceae, Pyrrophyceae, and zooplankton, which are important sources of lipids and fatty acids (i.e., PUFAs) in the gonads. These nutrients generally accumulated in the gonads during the development stage and finally translocated to the gametes during the maturation stages, which were afterwards metabolized to provide energy and structural materials during embryogenesis and early larval development. From February to April, *P. viridis* started to spawn, which was mainly triggered by the increasing temperature from its minimum level in winter, high salinity, and DO level. At that time, mature spermatozoa and ova were released, the gonadal content of lipids was decreased, and the gonad reached a quiescent state. In contrast, heavy rainfall and surface runoff decreased the salinity and food availability during the summer and monsoon seasons (May–September), while the temperature remained very high along the coastal regions of Bangladesh. These ecological factors prolonged the resting stage of *P. viridis*. Finally, we can say that this innovative research will provide a model toward a better understanding of the reproductive biological traits of the species based on the adequate knowledge needed for the sustainable exploitation of the fishery resources.

## Supplementary Materials

The following are available online at https://www.mdpi.com/2079-7737/8/4/88/s1, Figure S1: Macroscopic temporal variation of gonad development of green mussel *Perna viridis* collected from the coastal waters of Bangladesh, Table S1: Annual variation of water quality parameters of the green mussel *Perna viridis* collection sites during November 2017 to October 2018, Table S2: Annual variation of the major groups of ingested plankton (absolute abundance) in the whole gut of the green mussel *Perna viridis*, Table S3: Abundance of the major groups of ingested plankton in the whole gut at different gonadal development stages of the green mussel *Perna viridis*, Table S4: Annual variation in fatty acid content (% of total fatty acids) at different stages of gonad development of the green mussel *Perna viridis*, Table S5: Variation in fatty acid content (% of total fatty acids) of gonads in different gonadal development stages of the green mussel *Perna viridis*, Table S6: Principal component analysis of the water quality parameters of the green mussel collection sites, Table S7: Principal component analysis of different month-wise ingested gut plankton of green mussel *Perna viridis*, Table S8: Principal component analysis of different gonadal stage-wise ingested gut plankton of green mussel *Perna viridis*, Table S9: Principal component analysis of different month-wise gonadal fatty acids contents of green mussel *Perna viridis*, Table S10: Principal component analysis of different gonad development stage-wise fatty acids contents of green mussel *Perna viridis*, Table S11: Principal component analysis (PCA): relationships among the environmental factors, ingested gut plankton, lipids, and fatty acid content of gonad and GSI of green mussel *Perna viridis.*
