# Supplementary material for: Reproductive Biology and Ecology of the Green Mussel Perna viridis: A Multidisciplinary Approach
Source: Biology (Basel). 2019 Nov 15;8(4):88. doi: 10.3390/biology8040088 (PMC6955735; doi:10.3390/biology8040088)
Supplement: Supplementary file 1 [file biology-08-00088-s001.pdf]

biology

MDPI

Reproductive biology and ecology of the green mussel *Perna viridis*: A multidisciplinary approach

Supplementary Material

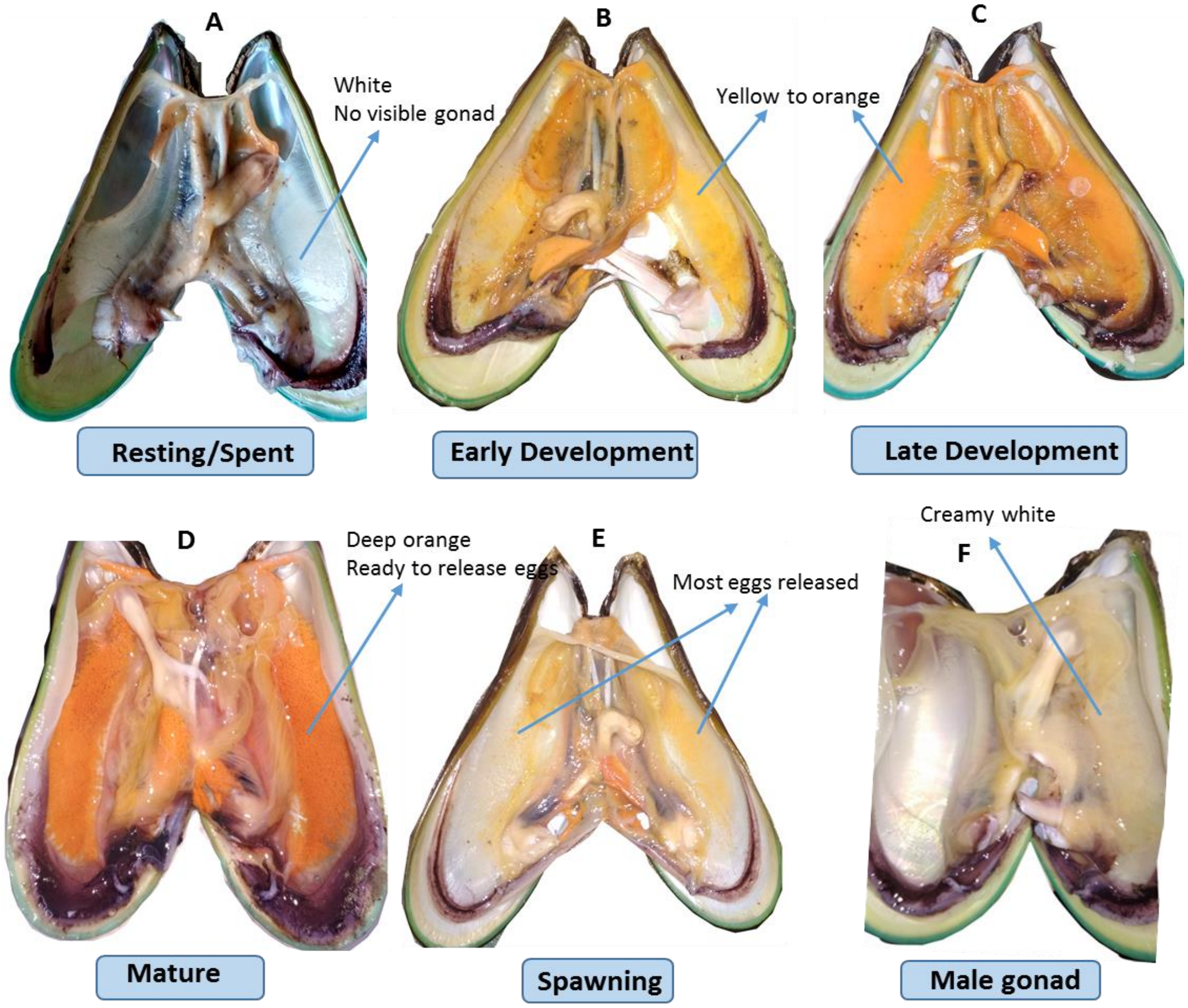

Figure S1. Macroscopic temporal variation of gonad development of green mussel *Perna viridis* collected from the coastal waters of Bangladesh. Female gonads color varied from white (resting) to yellow (early developmental) to deep orange (mature) stage. Males gonads are creamy white color during development stages but pictorial views are difficult to separate at different gonad development stage

Table S1. Annual variation of water quality parameters of the green mussel *Perna viridis* collection sites during November 2017 to October 2018.

Water Parameters	Annual variation												F-value	Sig. P-Value
	Jan	Feb	Mar	Apr	May	Jun	Jul	Aug	Sep	Oct	Nov	Dec		
Water depth (m)	6.4±0.06^{d}	7.0±0.06^{f}	7.3±0.06^{g}	7.6±0.06^{h}	5.6±0.06^{b}	5.4±0.02^{ab}	7.1±0.10^{fg}	6.8±0.01^{e}	6.5±0.02^{d}	5.2±0.12^{a}	6.1±0.06^{c}	6.1±0.09^{c}	387.81	0.000***
Temp. (^{0}C)	24.5±0.21^{c}	25.5±0.25^{d}	26.3±0.11^{e}	27.1±0.15^{f}	29.4±0.06^{h}	29.7±0.11^{h}	30.8±0.06^{i}	32.6±0.20^{g}	31.8±0.06^{k}	28.3±0.11^{e}	23.3±0.12^{b}	22.2±0.06^{a}	1727.89	0.000***
Curr speed (m/s)	0.35±0.01^{ab}	0.30±0.02^{a}	0.35±0.03^{ab}	0.35±0.02^{ab}	0.45±0.03^{d}	0.49±0.02^{de}	0.67±0.02^{f}	0.83±0.02^{g}	0.68±0.01^{f}	0.54±0.02^{e}	0.44±0.03^{cd}	0.37±0.01^{bc}	178.21	0.000***
Transparency(cm)	43.9±1.4^{abc}	51.6±2.4^{cde}	53.2±1.1^{de}	51.5±4.7^{cde}	66.7±4.8^{g}	63.7±5.02^{fg}	42.5±3.70^{ab}	53.2±1.11^{de}	56.6±1.64^{ef}	37.9±1.94^{a}	46.4±1.9^{abcd}	49.9±2.1^{bcde}	22.93	0.000***
pH	7.45±0.02^{e}	6.86±0.01^{b}	6.93±0.01^{b}	7.09±0.01^{c}	7.43±0.01^{e}	6.72±0.01^{a}	7.09±0.01^{e}	7.15±0.01^{cd}	7.20±0.01^{d}	6.92±0.01^{b}	7.67±0.10^{f}	7.18±0.01^{cd}	235.03	0.000***
DO (ppm)	7.57±0.32^{d}	7.66±0.13^{d}	6.86±0.35^{c}	6.90±0.10^{c}	6.80±0.10^{c}	6.23±0.10^{a}	6.32±0.01^{ab}	6.6±0.01^{abc}	6.25±0.01^{ab}	6.80±0.01^{c}	6.70±0.10^{bc}	6.90±0.10^{c}	25.29	0.000***
Salinity (ppt)	33.6±0.37^{k}	33.6±0.20^{k}	33.3±0.17^{jk}	32.0±0.21^{gh}	31.5±0.26^{g}	28.7±0.12^{e}	27.6±0.20^{d}	18.6±0.20^{a}	22.6±0.20^{b}	26.6±0.20^{c}	30.2±0.40^{f}	32.6±0.15^{ij}	1185.02	0.000***
Alkalinity (ppm)	135±6.2	135±12.2	138±13.2	134±20.5	135±11.4	132±16.8	141±20.1	136±17.5	129±15.5	124±21.1	135±8.9	137±25.4	0.19	0.996NS
NO_3-N (ppm)	0.52±0.04^{d}	0.41±0.04^{cd}	0.37±0.05^{bcd}	0.27±0.05^{abc}	0.23±0.04^{ab}	0.15±0.03^{a}	0.37±0.05^{bcd}	0.38±0.04^{bcd}	0.73±0.10^{e}	0.51±0.06^{d}	0.45±0.03^{d}	0.47±0.09^{d}	0.24	0.000***
PO_4-P (ppm)	1.34±0.04^{de}	1.07±0.04^{bcd}	0.84±0.15^{ab}	0.97±0.1^{abcd}	0.73±0.13^{ab}	0.68±0.09^{a}	1.29±0.20^{de}	0.89±0.08^{abc}	0.73±0.06^{ab}	1.76±0.11^{f}	1.47±0.16^{ef}	1.24±0.23^{cde}	21.80	0.000***
NH_3-N (ppm)	0.26±0.02^{de}	0.24±0.03^{cd}	0.21±0.03^{bcd}	0.24±0.01^{cd}	0.19±0.02^{bc}	0.32±0.01^{f}	0.31±0.01^{ef}	0.31±0.01^{ef}	0.21±0.01^{bcd}	0.23±0.01^{bcd}	0.12±0.01^{a}	0.18±0.01^{b}	32.13	0.000***
NH_4-N (ppm)	0.12±0.01	0.09±0.01	0.33±0.41	0.09±0.01	0.09±0.01	0.08±0.01	0.07±0.01	0.09±0.01	0.09±0.01	0.14±0.02	0.1±0.02	0.09±0.01	1.06	0.429 NS
Chlo-a (µg/L)	6.92±0.17^{fg}	5.81±0.24^{cde}	5.21±0.08^{bcd}	4.91±0.65^{abc}	4.32±0.49^{ab}	4.01±0.22^{a}	7.42±0.47^{gh}	5.33±0.09^{bcd}	4.55±0.28^{ab}	8.17±0.37^{h}	6.57±0.29^{efg}	5.98±0.36^{def}	40.88	0.000***
Plankton (×10^3/L)	70.6±3.51^{fg}	58.6±3.52^{cde}	53.7±2.52^{bcd}	48.3±5.15^{abc}	43.6±3.78^{ab}	41.7±3.51^{a}	76.0±5.29^{gh}	54.0±2.01^{bcd}	48.0±3.01^{ab}	88.7±3.05^{h}	68.0±4.0^{efg}	63.0±1.73^{def}	49.04	0.000***

Values are mean ± SD of triplicate measurements for each months based on the repeated measures ANOVA. The mean values followed by the different superscript letter in each parameter indicate significant difference at 0.05. If the effects were significant, ANOVA was followed by Tukey test. P-values are given while significance levels are denoted by asterisks (*<0.05; **<0.01; ***<0.001; NS-not significant).

Table S2. Annual variation of the major groups of ingested plankton (absolute abundance) in the whole gut of the green mussel *Perna viridis*

Gut Plankton Groups	Annual variation												F-value	Sig. level P-Value
	Jan	Feb	Mar	Apr	May	Jun	Jul	Aug	Sep	Oct	Nov	Dec		
Bacillariophyceae ($\times10^4$)	24.67 ± 9.7^{de}	23.7 ± 7.3^{d}	22.0 ± 0.005^{cd}	16.86 ± 4.9^{b}	10.27 ± 3.2^{a}	15.6 ± 8.2^{b}	16.6 ± 9.4^{b}	18.4 ± 8.2^{b}	22.8 ± 3.5^{cd}	35.7 ± 2.3^{e}	38.6 ± 9.02^{f}	28.1 ± 2.45^{e}	115.89	0.000***
Chlorophyceae ($\times10^4$)	1.47 ± 1.4^{abc}	1.40 ± 1.1^{abc}	1.9 ± 1.3^{c}	0.76 ± 1.4^{abc}	1.55 ± 1.4^{abc}	1.1 ± 1.4^{abc}	0.5 ± 0.8^{ab}	1.8 ± 1.3^{bc}	0.9 ± 1.2^{abc}	0.48 ± 0.87^{a}	0.5 ± 1.2^{ab}	0.84 ± 1.2^{abc}	3.47	0.000***
Cyanophyceae ($\times10^4$)	2.8 ± 2.6^{ab}	4.1 ± 2.6^{ab}	4.82 ± 2.81^{ab}	2.48 ± 2.75^{ab}	3.09 ± 2.74^{ab}	2.7 ± 2.85^{ab}	1.5 ± 1.93^{a}	5.5 ± 5.46^{b}	3.8 ± 7.28^{ab}	2.19 ± 2.18^{ab}	1.7 ± 2.54^{a}	2.84 ± 2.77^{ab}	2.40	0.008**
Dinophyceae ($\times10^4$)	2.4 ± 1.72^{bc}	2.2 ± 1.45^{abc}	2.91 ± 1.48^{c}	1.15 ± 1.49^{ab}	1.73 ± 1.67^{abc}	1.9 ± 1.77^{abc}	0.6 ± 1.47^{a}	0.8 ± 1.64^{ab}	1.5 ± 1.57^{abc}	0.86 ± 1.74^{ab}	1.4 ± 1.96^{abc}	2.42 ± 1.43^{bc}	4.12	0.000***
Pyrrophyceae	0.93 ± 1.28^{a}	2.0 ± 2.25^{ab}	1.55 ± 1.63^{ab}	2.09 ± 2.79^{ab}	1.18 ± 1.59^{ab}	0.6 ± 1.47^{a}	1.0 ± 1.99^{a}	2.1 ± 1.65^{ab}	1.8 ± 2.14^{ab}	3.24 ± 4.12^{ab}	3.9 ± 4.18^{b}	3.26 ± 4.17^{ab}	2.95	0.001***
Phytoplankton ($\times10^4$)	32.27 ± 4.65^{cd}	33.4 ± 5.15^{cd}	33.18 ± 4.69^{cd}	23.33 ± 7.7^{ab}	17.82 ± 6.64^{a}	21.9 ± 4.28^{ab}	20.2 ± 5.15^{a}	28.6 ± 6.46^{bc}	30.8 ± 10.0^{cd}	42.5 ± 5.51^{ef}	46.1 ± 10.18^{f}	37.47 ± 6.14^{de}	35.53	0.000***
Zooplankton ($\times10^4$)	4.4 ± 1.72^{ab}	4.6 ± 4.45^{ab}	3.81 ± 2.54^{ab}	3.62 ± 5.0^{ab}	3.55 ± 2.3^{a}	3.0 ± 2.94^{a}	4.3 ± 3.57^{ab}	4.3 ± 1.75^{ab}	7.1 ± 7.75^{ab}	7.9 ± 5.88^{b}	6.4 ± 3.53^{ab}	5.16 ± 3.29^{ab}	2.79	0.002**
Plankton ($\times10^4$)	36.67 ± 4.82^{cd}	38.0 ± 8.49^{d}	37.0 ± 5.68^{d}	26.95 ± 10.9^{abc}	31.36 ± 7.23^{a}	24.9 ± 3.86^{ab}	24.5 ± 8.28^{ab}	32.9 ± 6.63^{bcd}	37.9 ± 1.75^{d}	50.4 ± 10.4^{ef}	52.5 ± 12.34^{f}	42.63 ± 13.3^{de}	23.14	0.000***

Values are mean ± SD of twenty measurements for each months (n=20) based on the one-way ANOVA. The mean values followed by the different superscript letter in each parameter indicate significant difference at 0.05. If the effects were significant, ANOVA was followed by Tukey test. P-values were given while significance levels are denoted by asterisks (*<0.05; **<0.01; ***<0.001; NS-not significant).

Table S3. Abundance of the major groups of ingested plankton in the whole gut at different gonadal development stages of the green mussel *Perna viridis*

**Gut Plankton Group**	**Gonadal Development Stages**					**F-values**	**Sig. Level P value**
	**Resting**	**Development**	**Mature**	**Spawning**	**Spent**		
Bacillariophyceae ($\times 10^4$)	17.47 ±2.04^{b}	34.5 ± 6.73^{e}	24.54 ± 0.9^{d}	22.18 ± 0.58^{c}	10.67 ± 2.84^{a}	269.44	0.000***
Chlorophyceae ($\times 10^4$)	1.22 ± 1.3^{ab}	0.7 ± 1.21^{a}	0.97 ± 1.21^{ab}	1.59± 1.37^{b}	1.2 ± 1.45^{ab}	2.93	0.022**
Cyanophyceae ($\times 10^4$)	3.27 ± 3.79	2.63 ± 4.66	2.86 ± 2.24	4.47 ± 2.91	2.67 ± 2.84	1.64	0.166 NS
Dinophyceae ($\times 10^4$)	1.09 ± 1.63^{a}	1.37 ± 1.86^{a}	2.38 ± 1.23^{b}	2.82 ± 1.64^{b}	1.4 ± 1.49^{a}	9.30	0.000***
Pyrrophyceae ($\times 10^4$)	1.42 ± 2.05^{a}	3.2 ± 3.95^{b}	1.95 ± 2.65^{ab}	1.65 ± 2.23^{ab}	1.47 ± 1.48^{a}	4.28	0.002**
Phytoplankton ($\times 10^4$)	24.46 ± 6.39^{b}	42.4 ± 9.02^{d}	32.7 ± 4.84^{c}	32.71 ± 5.32^{c}	17.4 ± 5.95^{a}	91.94	0.000***
Zooplankton ($\times 10^4$)	4.1 ± 3.5^{a}	7.3 ± 5.88^{b}	4.54 ± 2.61^{a}	4.18 ± 3.86^{a}	3.0 ± 2.33^{a}	7.94	0.000***
Total Plankton ($\times 10^4$)	28.56 ± 8.19^{b}	49.7 ± 12.83^{d}	47.24 ± 6.7^{c}	36.88 ± 7.53^{c}	20.4 ± 6.33^{a}	68.77	0.000***

Values are mean ± SD. The mean values followed by the different superscript letter in each parameter indicate significant difference at 0.05. If the effects were significant, ANOVA was followed by Tukey test. P-values were given while significance levels were denoted by asterisks (*<0.05; **<0.01; ***<0.001; NS-not significant).

Table S4. Annual variation in fatty acid content (% of total fatty acids) at different stages of gonad development of the green mussel *Perna viridis*

Fatty Acids	Annual variation												F value	Sig. level P-Value
	Jan	Feb	Mar	Apr	May	Jun	Jul	Aug	Sep	Oct	Nov	Dec		
C14:0	3.24±0.26^{ab}	3.03±0.16^{ab}	3.08±0.31^{ab}	3.32±0.48^{ab}	3.65±0.29^{b}	3.41±0.25^{ab}	2.99±0.02^{ab}	3.25±0.55^{ab}	2.6±0.15^{a}	2.96±0.18^{ab}	2.96±0.12^{ab}	3.15±0.11^{ab}	2.701	0.02*
C15:0	0.61±0.08	0.58±0.07	0.69±0.06	0.71±0.11	0.59±0.14	0.61±0.08	0.63±0.08	0.51±0.08	0.59±0.05	0.62±0.05	0.60±0.12	0.76±0.05	1.91	0.09NS
C16:0	25.02±0.19^{d}	24.26±0.2^{cd}	21.68±3.42^{abcd}	18.9±3.93^{abcd}	15.6±1.03^{a}	16.57±1.81^{ab}	17.46±0.67^{abc}	17.19±1.16^{abc}	17.45±3.19^{abc}	19.4±0.52^{abcd}	21.37±4.1^{abcd}	23.81±4.17^{bcd}	4.85	0.001***
C17:0	0.9±0.07^{a}	0.98±0.05^{a}	1.10±0.21^{ab}	1.31±0.31^{ab}	1.44±0.10^{ab}	1.29±0.22^{ab}	1.15±0.38^{ab}	1.77±0.12^{b}	1.42±0.5^{ab}	1.12±0.16^{ab}	1.05±0.03^{a}	0.86±0.08^{a}	3.74	0.003**
C18:0	2.43±0.38^{a}	2.9±0.3^{ab}	3.06±0.44^{ab}	3.31±0.33^{abc}	3.63±0.04^{abc}	3.46±0.73^{abc}	3.07±0.37^{ab}	4.72±0.4^{c}	4.06±0.13^{bc}	3.62±0.52^{abc}	2.95±0.08^{ab}	2.35±0.45^{a}	4.49	0.001***
C20:0	0.29±0.06	0.34±0.06	0.39±0.06	0.39±0.09	0.34±0.07	0.34±0.23	0.39±0.02	0.44±0.21	0.28±0.1	0.39±0.09	0.32±0.04	0.36±0.03	0.89	0.567NS
C24:0	0.4±0.03^{ab}	0.36±0.11^{ab}	0.29±0.06^{ab}	0.31±0.03^{ab}	0.28±0.03^{ab}	0.38±0.13^{ab}	0.30±0.03^{ab}	0.34±0.12^{a}	0.33±0.08^{ab}	0.44±0.11^{ab}	0.35±0.1^{ab}	0.41±0.11^{ab}	1.02	0.458NS
Total SFAs	32.9±0.2^{d}	32.45±0.1^{cd}	30.3±2.5^{abcd}	28.24±2.9^{abcd}	25.56±0.69^{a}	26.06±0.9^{ab}	26.0±0.28^{ab}	28.2±0.95^{abcd}	26.7±1.72^{abc}	28.55±0.1^{abcd}	29.59±3.9^{abcd}	31.7±3.66^{bcd}	4.90	0.001***
C14:1	0.24±0.07	0.31±0.06	0.25±0.06	0.39±0.06	0.34±0.03	0.32±0.12	0.4±0.1	0.32±0.07	0.3±0.1	0.25±0.02	0.3±0.02	0.24±0.07	1.68	0.141NS
C16:1	10.07±0.43^{a}	9.98±0.21^{a}	12.29±3.51^{abc}	13.4±3.09^{abc}	15.86±0.8^{bc}	16.27±0.59^{c}	16.19±0.77^{c}	16.33±0.06^{c}	14.54±2.4^{abc}	11.42±0.27^{ab}	10.64±0.62^{a}	10.39±0.57^{a}	8.13	0.000***
C17:1	2.15±0.41	2.61±0.13	2.94±0.44	2.89±0.28	3.17±0.15	2.74±0.48	2.8±0.42	3.15±0.17	2.89±0.67	2.22±0.16	2.51±0.21	2.35±0.41	2.55	0.076NS
C18:1n-9	3.22±0.00^{a}	4.15±0.46^{abc}	4.08±1.58^{abc}	4.75±1.34^{abc}	5.55±0.39^{abc}	6.28±0.86^{c}	5.84±0.3^{bc}	5.68±0.33^{bc}	4.87±1.01^{abc}	4.11±0.25^{abc}	4.34±0.63^{abc}	3.84±0.92^{ab}	3.97	0.002**
C18:1n-7	2.1±0.21^{ab}	2.39±0.31^{abc}	2.69±0.22^{bc}	2.54±0.22^{abc}	2.59±0.22^{abc}	2.82±0.17^{c}	2.92±0.19^{c}	2.77±0.15^{bc}	2.55±0.34^{abc}	1.9±0.11^{a}	2.25±0.22^{abc}	2.1±0.33^{ab}	5.64	0.000***
C20:1n-9	1.9±0.36^{a}	2.8±0.31^{ab}	1.74±0.35^{a}	2.54±0.64^{ab}	2.41±0.14^{ab}	2.98±0.42^{b}	3.06±0.44^{b}	2.32±0.14^{ab}	2.41±0.19^{ab}	1.9±0.18^{a}	2.18±0.27^{ab}	1.89±0.36^{a}	4.49	0.001***
Total MUFAs	19.7±0.05^{a}	21.5±0.14^{ab}	23.99±4.75^{abc}	26.5±4.76^{abcd}	29.93±1.67^{cd}	31.43±0.06^{d}	31.21±0.13^{cd}	30.57±0.34^{cd}	27.55±4.4^{bcd}	21.83±0.14^{ab}	22.23±0.84^{ab}	20.82±0.1.49^{ab}	9.66	0.000***
C18:3n-3	2.0±0.55	1.65±0.43	2.25±0.15	1.86±0.28	1.31±0.38	1.31±0.13	1.54±0.5	1.67±0.44	1.93±0.74	2.4±0.12	2.1±0.08	2..07±0.13	2.43	0.081NS
C18:4n-3	2.87±0.34^{a}	2.83±0.53^{a}	3.37±0.22^{ab}	2.55±0.33^{a}	2.69±1.24^{a}	5.45±0.71^{c}	5.05±0.17^{bc}	5.73±0.26^{c}	4.48±1.53^{abc}	2.88±0.6^{a}	2.71±0.44^{a}	2.51±0.34^{a}	9.45	0.000***
C20:5n-3	16.6±0.6^{bcde}	16±0.7^{abcde}	16.39±0.79^{bcde}	15.5±1.0^{abcde}	14.8±0.53^{abcd}	11.9±1.15^{ab}	12.56±2.18^{ab}	11.23±1.18^{a}	14.39±4.57^{abc}	20.08±0.18^{e}	19.7±1.3^{de}	18.3±1.57^{cde}	8.37	0.000***
C22:5n-3	1.07±0.31^{ab}	0.72±0.42^{ab}	0.47±0.31^{a}	0.77±0.12^{ab}	0.95±0.42^{ab}	1.09±0.19^{ab}	0.98±0.14^{ab}	0.13±0.3^{b}	0.95±0.17^{ab}	0.55±0.39^{ab}	0.76±0.09^{ab}	0.87±0.02^{ab}	2.22	0.05*
C22:6n-3	12.2±0.23^{ab}	12.47±0.3^{b}	10.26±0.16^{ab}	11.35±1.07^{ab}	10.58±2.05^{ab}	8.84±1.34^{a}	8.8±1.2^{a}	10.15±0.99^{ab}	10.69±2.25^{ab}	12.81±0.66^{b}	12.9±1.5^{b}	12.94±0.4^{ab}	4.69	0.001***
n-3PUFAs	34.7±0.4^{bcd}	33.6±0.1^{abcd}	32.74±0.71^{abc}	32.0±0.11^{abc}	30.34±1.34^{ab}	28.6±0.28^{a}	28.9±1.47^{a}	30.08±0.15^{ab}	32.45±5.06^{abc}	38.7±0.8^{d}	38.2±1.9^{d}	36.7±1.5^{cd}	11.46	0.000***
C18:2n-6	0.7±0.11	1.03±0.56	1.3±0.36	1.32±0.33	1.34±0.14	1.17±0.26	1.09±0.26	1.42±0.26	1.05±0.18	1.11±0.13	1.06±0.38	0.86±0.07	1.56	0.175NS
C18:3n-6	0.24±0.04	0.23±0.1	0.4±0.06	0.42±0.15	0.33±0.1	0.39±0.04	0.33±0.05	0.37±0.04	0.35±0.06	0.3±0.09	0.24±0.1	0.28±0.04	2.01	0.074NS
C20:2n-6	1.03±0.13	1.01±0.48	1.14±0.39	1.33±0.68	1.6±0.45	1.57±0.53	1.56±0.6	2.04±0.14	1.88±0.87	1.34±0.47	1.28±0.20	1.07±0.13	1.45	0.216NS
C20:3n-6	0.37±0.08^{a}	0.39±0.12^{a}	0.61±0.05^{ab}	0.7±0.07^{ab}	0.62±0.23^{ab}	0.67±0.06^{ab}	0.67±0.05^{ab}	0.65±0.11^{ab}	0.63±0.11^{ab}	0.78±0.1^{b}	0.81±0.26^{b}	0.34±0.03^{a}	4.54	0.001***
C20:4n-6	8.63±0.83^{b}	8.21±1.8^{b}	7.27±2.03^{ab}	7.14±2.09^{ab}	7.75±1.16^{ab}	8.14±0.78^{b}	8.3±1.96^{b}	3.86±1.48^{a}	6.9±0.95^{ab}	5.35±1.06^{ab}	4.79±0.94^{ab}	6.37±0.52^{ab}	3.53	0.005**
C22:3n-6	0.49±0.1	0.37±0.1	0.64±0.05	0.63±0.17	0.69±0.15	0.62±0.16	0.55±0.08	0.55±0.07	0.65±0.18	0.58±0.22	0.59±0.26	0.47±0.05	1.10	0.401NS
C22:2n-6	1.21±0.22^{a}	1.21±0.24^{a}	1.6±0.24^{ab}	1.7±0.35^{ab}	1.8±0.56^{ab}	1.35±0.12^{ab}	1.34±0.3^{ab}	2.22±0.11^{b}	1.82±0.67^{ab}	1.44±0.05^{ab}	1.2±0.3^{a}	1.39±0.34^{ab}	2.75	0.018*
n-6 PUFAs	12.67±0.1^{ab}	12.45±0.3^{ab}	13.0±1.67^{ab}	13.25±1.86^{ab}	14.16±1.1^{b}	13.91±0.67^{b}	13.86±1.1^{b}	11.12±1.39^{ab}	13.27±2.42^{ab}	10.91±0.61^{ab}	9.98±0.32^{a}	10.78±0.73^{ab}	3.87	0.003***
Total PUFAs	47.4±0.25^{cde}	46.0±0.2^{bcde}	45.7±2.25^{abcde}	45.3±2.0^{abcde}	44.5±1.44^{abcd}	42.51±0.86^{ab}	42.79±0.41^{abc}	41.2±1.26^{a}	45.7±2.7^{abcde}	49.6±0.21^{e}	48.18±2..17^{de}	47.47±2.18^{cde}	7.28	0.000***
n.3:n.6	2.75±0.16^{abc}	2.7±0.08^{abc}	2.55±0.30^{abc}	2.45±0.32^{ab}	2.15±0.22^{a}	2.06±0.09^{a}	2.1±0.28^{a}	2.7±0.35^{abc}	2.56±0.93^{abc}	3.56±0.28^{cd}	3.8±0.13^{d}	3.4±0.13^{bcd}	8.21	0.000***

Values are mean ± SD of triplicate measurements (n=3) based on the one-way ANOVA. The mean values followed by the different superscript letter in each parameter indicate significant difference at 0.05. If the effects were significant, ANOVA was followed by Tukey test. P-values were given while significance levels were denoted by asterisks (*<0.05; **<0.01; ***<0.001; NS-not significant).

Table S5. Variation in fatty acid content (% of total fatty acids) of gonad in different gonadal development stages of the green mussel *Perna viridis*

Fatty acids	Gonadal Development Stages					F-values	Sig. Level P value
	Resting	Development	Mature	Spawning	Spent		
C14:0	3.17±0.48^{ab}	2.95±0.16^{a}	3.18±0.21^{ab}	2.96±0.14^{a}	3.51±0.17^{b}	2.88	0.03**
C15:0	0.59±0.09	0.64±0.07	0.65±.12	0.64±0.09	0.62±0.13	0.51	0.728NS
C16:0	16.62±1.34^{a}	19.47±0.81^{b}	25.6±0.67^{d}	23.9±0.39^{c}	16.66±0.74^{a}	127.81	0.000***
C17:0	1.47±0.33^{b}	1.03±0.14^{a}	0.9±0.1^{a}	0.98±0.04^{a}	1.41±0.11^{b}	11.37	0.000***
C18:0	3.92±0.86^{c}	3.18±0.53^{abc}	2.4±0.38^{a}	2.88±0.2^{ab}	3.57±0.09^{bc}	7.95	0.000***
C20:0	0.36±0.12	0.36±0.07	0.32±0.05	0.34±0.06	0.38±0.08^{b}	0.39	0.816 NS
C24:0	0.33±0.09^{ab}	0.44±0.08^{b}	0.36±0.07^{ab}	0.34±0.07^{ab}	0.28±0.03^{a}	3.89	0.011**
Total SFAs	26.46±1.24^{a}	28.08±0.64^{b}	33.41±0.58^{c}	32.07±0.44^{c}	26.43±0.82^{a}	90.30	0.000***
C14:1	0.35±0.09	0.28±0.04	0.23±0.05	0.31±0.08	0.33±0.07	3.01	0.053 NS
C16:1	16.25±0.46^{d}	11.28±0.33^{b}	10.05±0.3^{a}	10.05±0.34^{a}	15.5±0.49^{c}	436.06	0.000***
C17:1	2.99±0.39^{c}	2.34±0.22^{ab}	2.26±0.38^{a}	2.9±0.34^{c}	2.82±0.28^{bc}	7.39	0.000***
C18:1n-9	5.86±0.51^{c}	4.3±0.44^{b}	3.36±0.39^{a}	3.67±0.62^{ab}	5.52±0.23^{c}	42.07	0.000***
C18:1n-7	2.82±0.16^{b}	2.12±0.23^{a}	2.08±0.25^{a}	2.55±0.29^{b}	2.5±0.17^{b}	17.91	0.000***
C20:1n-9	2.7±0.43^{c}	2.12±0.25^{ab}	1.89±0.31^{a}	1.86±0.33^{a}	2.52±0.37^{bc}	8.87	0.000***
Total MUFAs	30.98±0.6^{e}	22.43±0.7^{c}	19.87±0.2^{a}	21.34±0.23^{b}	29.18±0.26^{d}	804.49	0.000***
C18:3n-3	1.5±0.49^{a}	2.21±0.19^{b}	2.07±0.36^{ab}	1.93±0.43^{ab}	1.75±0.3^{ab}	4.49	0.006**
C18:4n-3	5.29±0.58^{a}	2.7±0.5^{b}	2.8±0.23^{b}	2.95±0.59^{b}	2.5±0.52^{b}	48.53	0.000***
C20:5n-3	12.1±1.6^{a}	19.97±0.74^{c}	17.26±1.0^{b}	16.02±0.86^{b}	15.49±0.72^{b}	53.67	0.000***
C22:5n-3	1.13±0.23^{b}	0.69±0.27^{a}	0.95±0.24^{ab}	0.63±0.37^{a}	0.7±0.06^{a}	6.19	0.001***
C22:6n-3	9.2±1.09^{a}	13.21±0.63^{c}	12.22±0.57^{bc}	11.64±1.17^{b}	11.17±0.87^{b}	23.99	0.000***
n-3 PUFAs	29.23±0.89^{a}	38.78±0.63^{e}	35.3±0.69^{d}	33.18±0.61^{c}	31.6±0.61^{b}	205.95	0.000***
C18:2n-6	1.24±0.25^{b}	1.09±0.18^{ab}	0.74±0.12^{a}	1.16±0.44^{ab}	1.3±0.25^{b}	4.32	0.007**
C18:3n-6	0.35±0.06^{a}	0.31±0.05^{a}	0.23±0.06^{b}	0.36±0.16^{b}	0.35±0.09^{ab}	2.82	0.061NS
C20:2n-6	1.86±0.48^{b}	1.23±0.35^{a}	1.07±0.11^{a}	0.97±0.32^{a}	1.51±0.47^{ab}	7.34	0.000***
C20:3n-6	0.67±0.1^{bc}	0.76±0.21^{c}	0.38±0.1^{a}	0.53±0.18^{ab}	0.59±0.1^{abc}	6.62	0.001***
C20:4n-6	6.85±2.19^{ab}	5.32±1.08^{a}	7.35±1.52^{ab}	8.42±1.21^{b}	6.83±1.79^{ab}	2.77	0.045**
C22:3n-6	0.59±0.11^{ab}	0.63±0.17^{ab}	0.45±0.1^{a}	0.48±0.17^{ab}	0.68±0.12^{b}	3.09	0.03**
C22:2n-6	1.79±0.5	1.38±0.22	1.2±0.14	1.51±0.43	1.52±0.21	2.91	0.053NS
n-6 PUFAs	13.33±1.6^{b}	10.72±0.64^{a}	11.42±1.46^{ab}	13.42±1.22^{b}	12.78±1.61^{ab}	5.61	0.002**
Total PUFAs	42.57±1.21^{a}	49.5±0.47^{d}	46.72±0.78^{c}	46.59±0.65^{c}	44.39±1.04^{b}	68.40	0.000***
n-3:n-6	2.23±0.36^{a}	3.63±0.26^{b}	3.14±0.46^{b}	2.49±0.26^{a}	2.5±0.34^{a}	21.28	0.000***

Values are mean ± SD of the gonadal fatty acids. The mean values followed by the different superscript letter in each parameter indicate significant difference at 0.05. If the effects were significant, ANOVA was followed by Tukey test. P-values were given while significance levels were denoted by asterisks (*<0.05; **<0.01; ***<0.001; NS-not significant).

Table S6. Principal component analysis of the water quality parameters of the green mussel collection sites. Eigenvalues, explained and cumulative variance, loadings of the variables for the first five PCs.

**Water Quality Parameters**	**Principal Components**		
	**PC1**	**PC2**	**PC3**
Variance explained			
Eigenvalues	4.47	3.09	1.55
% of variance	40.68	28.10	14.05
Cumulative %	40.68	68.78	82.83
Factor loadings			
Temperature	-0.731	0.586	-0.062
Current speed	-0.460	0.842	0.193
Transparency	-0.794	-0.487	0.175
pH	0.336	-0.066	0.767
DO	0.590	-0.457	-0.204
Salinity	0.475	-0.802	-0.230
Nitrate-nitrogen	0.376	0.392	0.506
Phosphate	0.865	0.349	-0.082
Ammonia-nitrogen	-0.444	0.391	-0.688
Chlorophyll-a	0.824	0.496	-0.177
Total plankton	0.804	0.520	-0.156

Table S7. Principal component analysis of different month-wise ingested gut plankton of green mussel *Perna viridis*. Eigenvalues, explained and cumulative variance, loadings of the variables for the first five PCs.

	Principal Components		
	**PC1**	**PC2**	**PC3**
Variance explained			
Eigenvalues	3.58	1.76	1.05
% of variance	44.79	22.04	13.07
Cumulative %	44.79	66.83	79.90
Factor loadings			
Bacillariophyceae	0.783	-0.343	0.210
Chlorophyceae	0.034	0.899	-0.089
Cyanophyceae	0.328	0.842	-0.206
Dinophyceae	0.074	0.249	0.884
Pyrophyceae	0.686	-0.191	-0.330
Phytoplankton	0.949	0.112	0.149
Zooplankton	0.705	-0.117	-0.188
Total plankton	0.994	0.052	0.059

Table S8. Principal component analysis of different gonadal stage-wise ingested gut plankton of green mussel *Perna viridis*. Eigenvalues, explained and cumulative variance, loadings of the variables for the first five PCs.

	**Principal Components**		
	**PC1**	**PC2**	**PC3**
Variance explained			
Eigenvalues	3.60	1.75	1.06
% of variance	45.01	21.92	13.31
Cumulative %	45.01	66.93	80.25
Bacillariophyceae	0.792	-0.327	0.226
Chlorophyceae	0.037	0.902	-0.082
Cyanophyceae	0.331	0.842	-0.202
Dinophyceae	0.083	0.226	0.879
Pyrophyceae	0.682	-0.205	-0.343
Phytoplankton	0.950	0.113	0.158
Zooplankton	0.708	-0.127	-0.218
Total plankton	0.994	0.051	0.057

Table S9. Principal component analysis of different month-wise gonadal fatty acids contents of green mussel *Perna viridis*. Eigenvalues, explained and cumulative variance, loadings of the variables for the first five PCs.

**Gonadal fatty acids**	**Principal Components**				
	**PC1**	**PC2**	**PC3**	**PC4**	**PC5**
Variance explained					
Eigenvalues	10.91	3.13	1.63	1.52	0.99
% of variance	51.96	14.91	7.74	7.23	4.71
Cumulative %	51.96	66.87	74.61	81.84	86.55
Factor loadings					
C14:0	0.262	0.047	-0.062	-0.626	0.621
C16:0	-0.817	-0.405	-0.333	-0.065	-0.160
C17:0	0.782	0.353	-0.202	0.235	0.228
C18:0	0.680	0.407	-0.155	0.443	0.143
Total SFAs	-0.757	-0.359	-0.487	-0.028	-0.068
C16:1	0.943	0.181	0.070	-0.107	0.064
C18:1n-9	0.870	0.259	0.141	-0.256	0.000
C18:1n-7	0.765	-0.236	0.043	0.087	-0.136
C20:1n-9	0.687	0.057	0.293	-0.430	-0.255
Total MUFAs	0.965	0.143	0.112	-0.131	0.003
C18:4n-3	0.774	-0.014	-0.301	0.011	-0.436
C20:5n-3	-0.852	0.343	0.260	0.022	0.051
C22:5n-3	0.484	-0.010	-0.498	-0.194	-0.031
C22:6n-3	-0.821	0.239	0.035	-0.014	0.224
n-3 PUFAs	-0.885	0.423	0.101	0.039	-0.022
C20:2n-6	0.282	0.552	0.437	0.166	-0.265
C20:4n-6	0.026	-0.894	0.354	0.076	0.086
C22:2n-6	0.527	0.212	-0.345	0.556	0.212
n-6 PUFAs	0.518	-0.671	0.325	0.332	0.156
Total PUFAs	-0.849	0.143	0.335	0.257	0.068
n-3:n-6	-0.729	0.628	-0.118	-0.157	-0.131

Table S10. Principal component analysis of different gonad development stage-wise fatty acids contents of green mussel *Perna viridis*. Eigenvalues, explained and cumulative variance, loadings of the variables for the first five PCs.

**Gonadal fatty acids**	**Principal Components**				
	**PC1**	**PC2**	**PC3**	**PC4**	**PC5**
Variance explained					
Eigenvalues	12.30	3.57	2.04	1.66	1.14
% of variance	47.30	13.73	7.83	6.37	4.39
Cumulative %	47.30	61.03	68.86	75.22	79.62
Factor loadings					
C14:0	0.273	0.036	-0.236	0.488	-0.622
C16:0	-0.819	-0.394	-0.287	-0.132	-0.028
C17:0	0.794	0.310	-0.182	-0.242	0.022
C18:0	0.680	0.360	-0.056	-0.336	0.249
C24:0	-0.414	0.072	0.287	0.423	0.413
Total SFAs	-0.759	-0.363	-0.417	-0.201	-0.036
C14:1	0.940	0.146	0.022	0.129	0.024
C16:1	0.587	-0.155	0.220	-0.439	-0.133
C18:1n-9	0.874	0.217	-0.044	0.258	-0.009
C18:1n-7	0.766	-0.235	0.145	-0.210	-0.035
C20:1n-9	0.678	0.043	0.107	0.487	0.080
Total MUFAs	0.965	0.109	0.052	0.135	0.006
C18:3n-3	-0.625	0.193	0.265	-0.344	0.174
C18:4n-3	0.754	-0.067	-0.227	-0.056	0.468
C20:5n-3	-0.831	0.390	0.170	0.042	-0.155
C22:5n-3	0.490	-0.058	-0.453	0.301	0.184
C22:6n-3	-0.816	0.250	-0.004	0.085	-0.049
n-3 PUFAs	-0.875	0.453	0.056	0.040	0.051
C18:2n-6	0.514	0.440	0.001	-0.336	-0.410
C20:2n-6	0.715	0.393	-0.241	-0.189	0.022
C20:3n-6	0.302	0.587	0.429	-0.068	-0.023
C20:4n-6	-0.010	-0.879	0.383	0.103	-0.002
C22:3n-6	0.286	0.363	0.608	0.176	-0.042
n-6 PUFAs	0.501	-0.631	0.467	-0.110	-0.064
Total PUFAs	-0.846	0.207	0.364	-0.016	0.028
n-3:n-6	-0.712	0.620	-0.226	0.068	0.078

Table S11. Principal Component Analysis (PCA): relationships among the environmental factors, ingested gut plankton, lipids and Fatty acid content of gonad and GSI of green mussel *Perna viridis*. Eigenvalues, explained and cumulative variance, loadings of the variables for the first five PCs.

**Gonadal fatty acids**	**Principal Components**		
	**PC1**	**PC2**	**PC3**
Variance explained			
Eigenvalues	10.29	3.43	2.12
% of variance	51.47	17.17	10.58
Cumulative %	51.47	68.64	79.22
Factor loadings			
Temperature	-0.806	0.474	-0.224
Current speed	-0.464	0.801	-0.185
Transparency	-0.687	-0.380	0.439
DO	0.514	-0.619	-0.296
Salinity	0.435	-0.749	0.245
Phosphate-phosphorus	0.795	0.331	-0.252
Chlorophyll-a	0.692	0.418	-0.368
Water planktons	0.704	0.450	-0.355
GSI	0.915	-0.299	-0.100
Gut Bacillariophyceae	0.751	0.186	0.555
Gut phytoplanktons	0.704	0.267	0.575
Gut zooplanktons	0.584	0.357	0.462
Gut planktons	0.717	0.312	0.581
Gonad SFAs	0.603	-0.462	-0.302
Gonad MUFAs	-0.820	0.384	0.226
Gonad n-3 PUFAs	0.870	0.036	-0.089
Gonad n-6 PUFAs	-0.621	-0.338	0.089
Gonad PUFAs	0.764	-0.163	-0.063
Gonad n-3:n-6	0.792	0.257	-0.058
Gonad total lipid	0.869	0.060	-0.044
